# Exploring the diversity of Brazilian camallanids: a checklist of the family Camallanidae (Nematoda) from Brazil with a new key to identification of the genera in the family

**DOI:** 10.1017/S0031182026101668

**Published:** 2026-04

**Authors:** Leandro Mauricio Oliveira Silva, Ana Nunes Santos, Luiz Felipe Ferreira Trindade, Francisco Tiago Vasconcelos Melo

**Affiliations:** 1 Laboratory of Cellular Biology and Helminthology ‘Profa. Dra. Reinalda Marisa Lanfredi’, Institute of Biological Sciences, Federal University of Pará (UFPA), Belém, Brazil; 2 Postgraduate Program in Tropical Biodiversity (PPGBIO), Federal University of Amapá (UNIFAP), Rod. Juscelino Kubitschek, km 02, Jardim Marco Zero, Macapá, AP CEP: 68903-419, Brazil

**Keywords:** Brazil, Camallanidae, checklist of Camallanidae, Nematoda, new key for Camallanidae

## Abstract

The family Camallanidae includes nematodes traditionally classified based on the morphology of their buccal capsules. However, several questions have been raised about the validity of these characteristics for their classification. Despite having a remarkable diversity, our knowledge of camallanids in Brazil remains limited, leaving gaps in our understanding of the true species diversity in the country, their geographical distribution and host species associations. Therefore, this study presents a checklist of species in the family Camallanidae recorded in Brazil, including a review for the classificationa and new dichotomous key for identifying the genera. Camallanidae comprises 2 subfamilies with 13 valid genera, classified based on the morphology of the buccal capsule and trident, and on the presence, shape and distribution of internal ridges on the capsule. Thirty-seven species, distributed across 7 genera, have been recorded in Brazil so far, parasitizing 276 host taxa, including fish, chelonians and snakes, with no records of these nematodes parasitizing amphibians in the country. We reallocated five species of *Spirocamallanus* and 2 species of *Procamallanus* to *Denticamallanus*, and 1 species of *Camallanus* was reallocated to *Serpinema. Spirocamallanus* is the most diverse genus, with 16 species, and *Spirocamallanus inopinatus* exhibited the highest host taxa association diversity (144) and the widest geographical distribution. Until further molecular studies are conducted, the new dichotomous key presented in this checklist contributes to a better understanding of the classification of the family Camallanidae, based on the morphology of the buccal capsule and accessory structures.

## Graphical abstract

**Figure:**
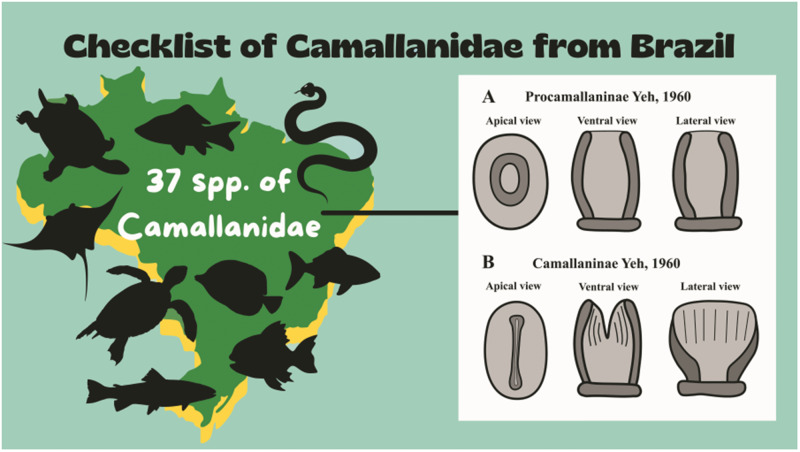

## Introduction

The family Camallanidae Railliet and Henry, 1915 (Nematoda: Rhabditida) (De Ley and Blaxter, 2002) includes parasitic nematodes that inhabit the gastrointestinal tracts of amphibians, fish and reptiles (Moravec, 1998). The representatives of this family are distinguished from other nematodes by 2 main characteristics: the reddish coloration of the body in life and the brown-orange, well-sclerotized buccal capsule (Rigby and Rigby, 2014).

The classification of genera within the family Camallanidae is based on the morphology of the buccal capsule, specifically on the presence, shape and distribution of internal ridges within the capsule, and on the trident morphology (Rigby and Rigby, 2014). Yeh (1960) divided the family into 2 subfamilies based on the buccal capsule structure: Procamallaninae Yeh, 1960, which features a continuous and rounded, cup-like buccal capsule, and Camallaninae Yeh, 1960, with a buccal capsule divided into 2 shell-like lateral valves.

The taxonomic history of the family Camallanidae is complex, with numerous genera proposed over the years. However, many of which have been synonymized or had their status questioned. These inconsistencies in the systematics of these genera arise from a diversity of variations in the buccal capsule morphology, particularly the internal ornamentations. These findings have led to some debate about the actual number of genera within Camallanidae (Svitin et al., 2019).

Despite the existence of checklists for nematode species parasitizing fish in Brazil (Luque et al., 2011) and specifically in the Brazilian Amazon biome (Santos Reis et al., 2021), and turtles in South America (Mascarenhas and Müller, 2021) and Neotropical region (Izidro de Brito and Figueiredo Lacerda, 2025), a focused and comprehensive survey of Camallanidae species specifically recorded in Brazil remains to be done.

The vast diversity of biomes and vertebrates in the country contributes to a high number of parasitic helminth species, including camallanids. There are several records of Camallanidae in Brazil (Mascarenhas and Müller, 2021; Santos Reis et al., 2021); however, the diversity of this group is still relatively unknown, as we do not have a compilation of its geographical distribution in the country or its distribution among host species.

The accurate species identification is fundamental for understanding parasite ecology, host specificity, geographic distribution and potential impacts on host health (Kholia and Fraser-Jinkins, 2011; Vink et al., 2012; Smales, 2022). Moreover, a checklist provides data for further investigations, highlighting unexplored regions or host groups and taxa that need to be investigated. At the same time, a taxonomic key is indispensable for both beginners and experienced parasitologists to identify parasites confidently. Without these tools, misidentifications can propagate, leading to flawed scientific conclusions and impeding the advancement of parasitological knowledge.

Thus, the objective of this study is to present a comprehensive checklist of Camallanidae species reported in Brazil and to provide a new, updated taxonomic key for the camallanid genera, based on a critical, revised understanding of their morphology and phylogenetic data.

## Material and methods

Bibliographical research on the Camallanidae species, their host records and geographical reports in Brazil was conducted by considering articles identified in the indexing databases Google Scholar, “Periódicos Capes-Brazil”, PubMed, SciELO and Scopus. The key terms used included ‘Camallanidae’ and related taxonomic names (at the genus level), combined with ‘Brazil’ and host-related keywords (‘fish’, ‘turtle’, ‘reptiles’, ‘Testudines’, 'amphibians'). Different combinations of these terms were tested (e.g. ‘Camallanidae Brazil’, ‘Camallanidae fish Brazil’, ‘Camallanidae turtle Brazil’) to retrieve all available records of Camallanidae nematodes parasitizing vertebrate hosts in the country.

Monographic works and abstracts presented at scientific events were not considered. Furthermore, we reviewed all records presented in checklists or similar works that present nematode surveys in Brazil: Vicente et al. (1985), Vicente et al. (1993), Moravec (1998), Vicente and Pinto (1999), Thatcher (2006), Luque et al. (2011), Eiras et al. (2016), Lehun et al. (2020), Mascarenhas and Müller (2021), Santos Reis et al. (2021), and Izidro de Brito and Figueiredo Lacerda (2025).

This review was based on a thorough reanalysis of all references cited by the authors, including checks of the species, hosts and localities reported in the original publications. When the original works were not accessible or the information conflicted with that presented in the checklists, such records were considered invalid. Thus, only confirmed records were considered in the results.

We also produce a map illustrating the geographical distribution of *Spirocamallanus inopinatus* (Travassos, Artigas and Pereira, 1928) in Brazil. The map was generated using a spreadsheet and QGIS 3.28 software (Quantum, 2024). The geographic coordinates, hosts and references used are presented in Supplementary Material 1.

To confirm the taxonomic status for some species, we examined by light microscopy the following camallanids specimens deposited in the Helminthological Collection of Oswaldo Cruz Institute (CHIOC)-Rio de Janeiro, Brazil: *Oncophora melanocephala* (Rudolphi, 1819) CHIOC 32248a-b, 32249 a-b, 33967; *Paracamallanus amazonensis* Ferraz and Thatcher, 1992 CHIOC 32945, 32948, 32956 e 32957; *Procamallanus petterae* Kohn and Fernandes, 1988 CHIOC 32430b; *Procamallanus* (*Procamallanus*) *peraccuratus* Pinto, Fabio, Noronha and Rolas, 1976 CHIOC 31084b-c; *Procamallanus* (*Spirocamallanus*) *intermedius* Pinto, Fabio, Noronha and Rolas, 1974 CHIOC 14658, 31022b-h, 31023a-c; *Procamallanus* (*Spirocamallanus*) *pimelodus* Pinto, Fabio, Noronha and Rolas, 1974 CHIOC 30993b-i, 30999b-f; and *Procamallanus* (*Spirocamallanus*) *saofranciscencis* (Moreira, Oliveira and Costa, 1994) CHIOC 37857, 37858.

The checklist is organized alphabetically by camallanid genera and species. The host species names were updated according to Froese and Pauly (2025) for fish and Uetz et al. (2025) for reptiles. We also provide records of camallanids in Brazil, analysing the status of the listed species and some taxonomic changes where applicable. Our records for each nematode taxon include any existing synonyms, host records, host habitat, sites of infection, locality records indicating Brazilian states, Brazilian biomes, deposit codes in helminthological collections (if available), references, unconfirmed reports from previous surveys and remarks. Remarks were presented when we analysed specimens, proposed relocating a species or discussed the species’ status.

The abbreviations for the helminthological collections reported in this study are: Collection of Non-Arthropod Invertebrates of the Museu Paraense Emílio Goeldi (MPEG), Helminthological Collection of Oswaldo Cruz Institute (CHIOC), Helminthological Collection of the Institute of Biosciences of Botucatu (CHIBB), Helminth Collection of the Laboratory of Parasitology of Wild Animals of the Federal University of Pelotas (CHLAPASIL-UFPel), Helminth Collection, University of Nebraska State Museum, Harold W. Manter Laboratory (HWML) and National Institute for Amazonian Research (INPA).

## Results

Based on the literature review and morphological analysis, we recognize only 2 subfamilies and 13 genera valid within Camallanidae. We followed 2 key conclusions proposed by Ailán-Choke and Pereira (2021) concerning the current classification of this family based on the buccal capsule morphology: (i) this classification remains consistent for some genera, reflecting morphological features congruent with the phylogeny of these nematodes; and (ii) it does not provide support for recognition of subgenera. Therefore, it is important to note that the subgeneric classification within Camallanidae should not be considered.

**Figure 1.**
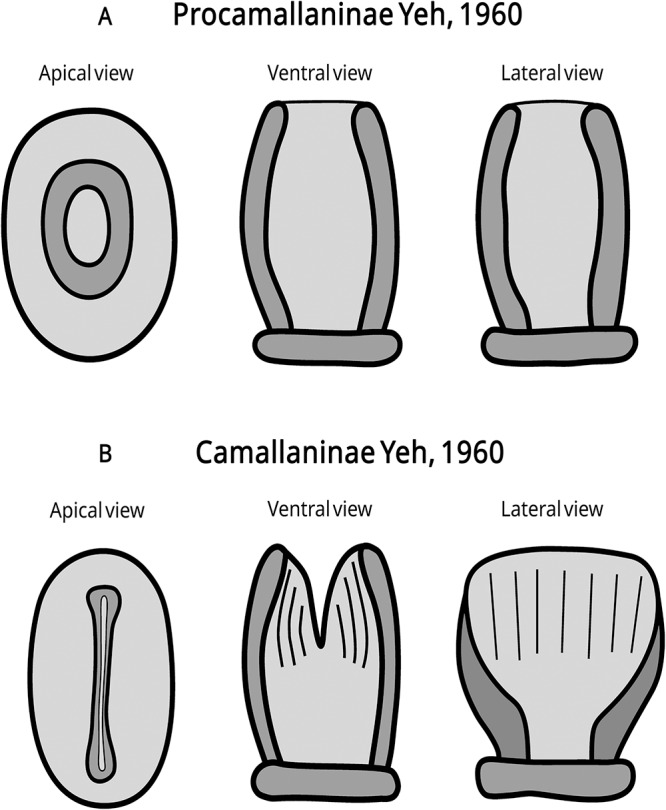
Illustrative drawing of buccal capsule morphology of the subfamilies within the family Camallanidae Railliet and Henry, 1915. (A) Procamallaninae; (B) Camallaninae. Figure 1 long description.

Based on a comprehensive literature review and our extensive analyses of camallanid specimens, the subfamilies were distinguished based on morphology of the buccal capsule (round without divisions vs. divided into 2 valves) (Fig. 1). For the genera, we used the presence and morphology of tridents (accessory structures on the buccal capsule), as well as the presence, morphology and distribution pattern of internal ornamentations in the buccal capsule (including spiral ridges, longitudinal ridges, punctations, ridges separated by a gap, tooth-like structures and a smooth internal surface). The new taxonomic identification key for camallanids is provided at the end of the article, including a diagnosis for Camallanidae, and diagnostic criteria for its subfamilies and genera.

**Figure 2.**
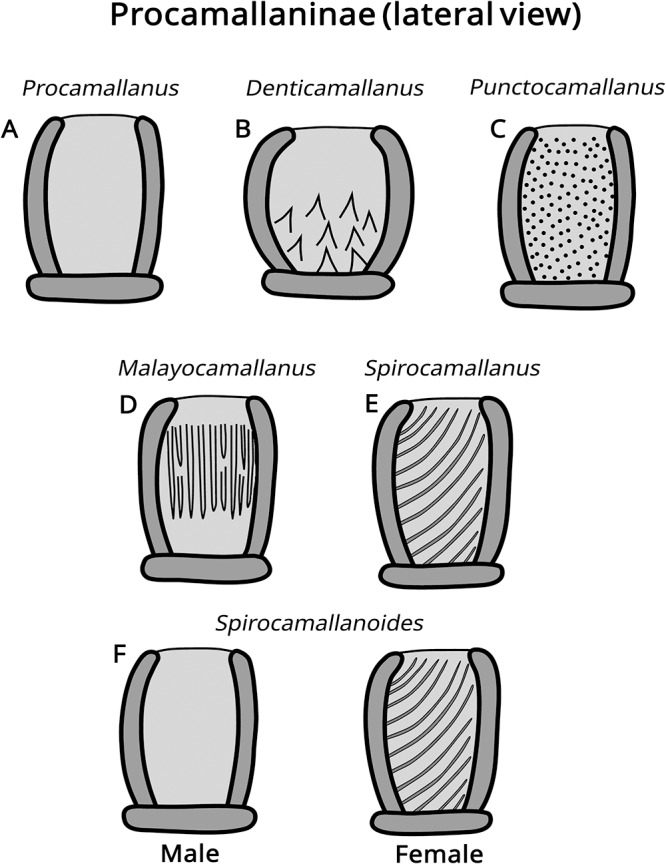
Illustrative drawings of buccal capsule morphology of the genera in the subfamily Procamallaninae Yeh, 1960. (A) *Procamallanus*; (B) *Denticamallanus*; (C) *Punctocamallanus*; (D) *Malayocamallanus*; (E) *Spirocamallanus*; (F) *Spirocamallanoides*. Figure 2 long description.

The subfamily Procamallaninae (buccal capsule rounded without divisions) includes the genera *Denticamallanus* Moravec and Thatcher, 1997, *Malayocamallanus* Jothy and Fernando, 1970, *Procamallanus* Baylis, 1922, *Punctocamallanus* Moravec and Scholz, 1991, *Spirocamallanus* Olsen, 1952, and *Spirocamallanoides* Moravec and Sey, 1988 (Fig. 2), distinguished by the presence/absence and morphology of internal ornamentations in the buccal capsule.

**Figure 3.**
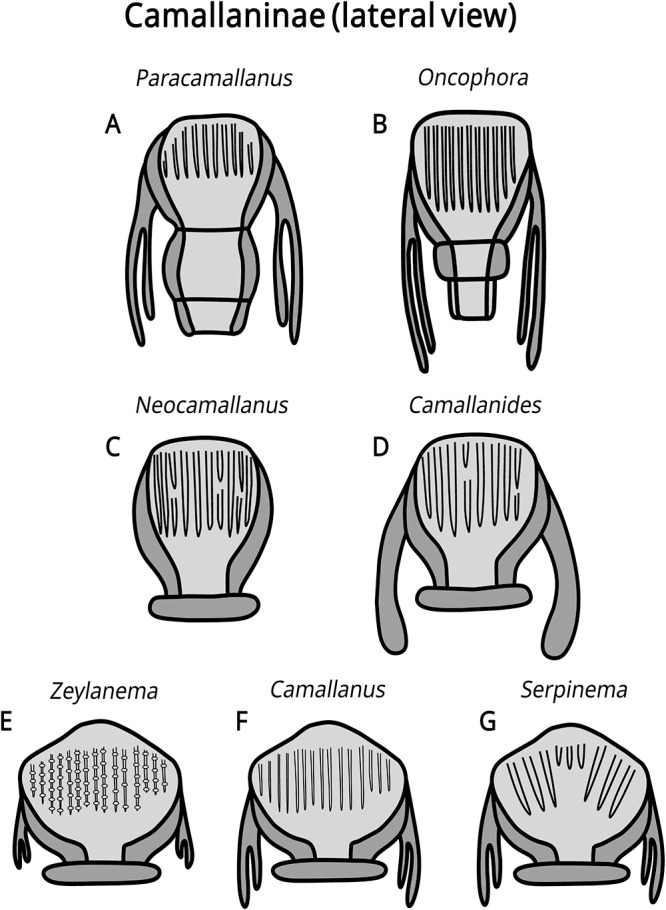
Illustrative drawings of buccal capsule morphology of the genera in the subfamily Camallaninae Yeh, 1960. (A) *Paracamallanus*; (B) *Oncophora*; (C) *Neocamallanus*; (D) *Camallanides*; (E) *Zeylanema*; (F) *Camallanus*; (G) *Serpinema*. Figure 3 long description.

**Figure 4.**
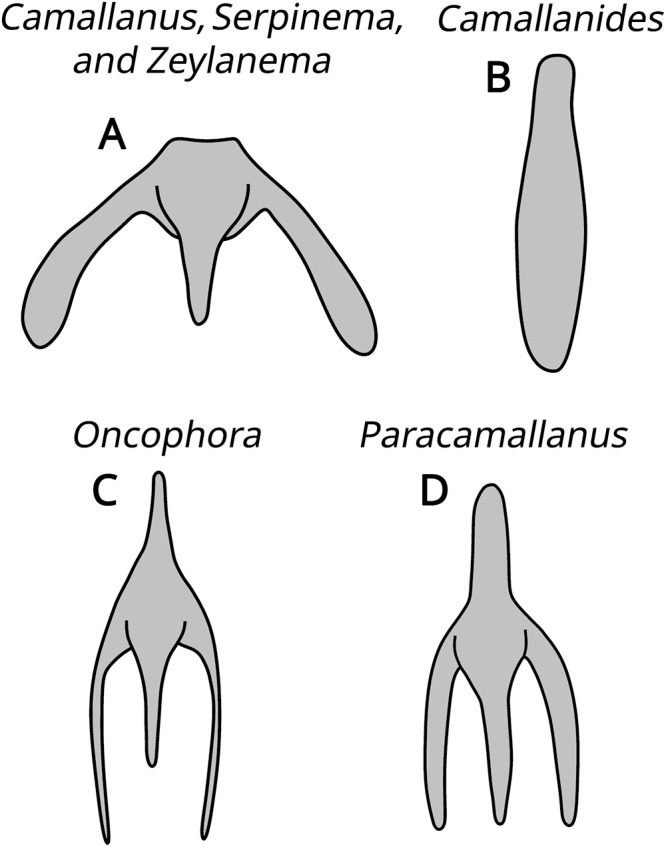
Illustrative drawings of the morphology of the tridents. (A) *Camallanus, Serpinema* and *Zeylanema*; (B) *Camallanides*; (C) *Oncophora*; (D) *Paracamallanus*. Figure 4 long description.

The subfamily Camallaninae (buccal capsule divided into 2 valves) is represented by the genera *Camallanides* Baylis and Daubney, 1922, *Camallanus* Railliet and Henry, 1915, *Neocamallanus* Ali, 1957, *Oncophora* Diesing, 1851, *Paracamallanus* Yorke and Maplestone, 1926, *Serpinema* Yeh, 1960, and *Zeylanema* Yeh, 1960 (Fig. 3). We can distinguish *Oncophora* and *Paracamallanus* because they have a buccal capsule divided into 3 chambers, as well as by the relative size of these chambers and the tridents morphology. *Camallanus, Serpinema* and *Zeylanema* are morphologically similar, sharing similar tridents, but we differentiate them by the morphology and distribution pattern of the internal ridges on the buccal capsule. *Camallanides* lacks tridents, whereas *Neocamallanus* possesses a trident composed of a single branch. Figure 4 illustrates the morphology of the tridents.

**Figure 5.**
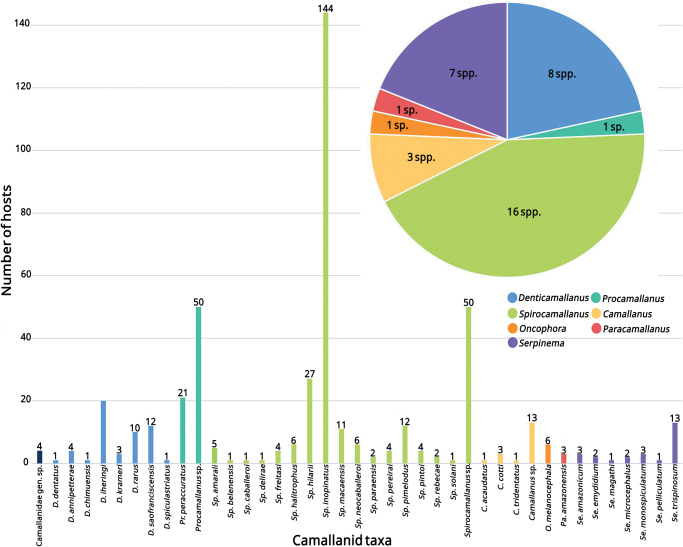
Diversity of Camallanidae taxa reported in Brazil, with the number of species per genus and the number of hosts per camallanid taxa. Figure 5 long description.

We identified 37 species of Camallanidae in Brazil, belonging to 7 genera. *Spirocamallanus* was the most diverse genus, with 16 species. The species *Sp. inopinatus* (Travassos, Artigas and Pereira, 1928) showed the highest number of host records (144 hosts). In contrast, *Procamallanus* was the least diverse genus, and we found 10 camallanid species parasitizing only a single host species (Fig. 5). In total, we found records of 276 host taxa, comprising 265 fish, 10 chelonians and 1 snake. Among the 265 fish hosts, some were identified only to order (1), family (2), subfamily (2) or genus (16); 1 taxon corresponds to a hybrid species (*Colossoma macropomum* × *Piaractus brachypomus*), and 2 taxa the authors were unable to confirm the species. Additionally, 17 fish hosts, not included in the count above, were identified only by their local names, preventing the determination of host species. We did not find any records of camallanids in Brazilian amphibians. We also provide a table with all host taxa, organized by families (Table 1).

**Table 1.** Records of vertebrate hosts of Camallanidae species reported in Brazil Table 1 long description.

Host species	Camallanidae species
**Fishes**	
**Siluriformes fam. gen. sp.**	*Procamallanus* sp.
**Acestrorhamphidae**	
*Astyanax altiparanae* Garutti and Britski, 2000	*Spirocamallanus caballeroi, Spirocamallanus inopinatus*
*Astyanax bimaculatus* (Linnaeus, 1758)	*Denticamallanus saofranciscensis, Procamallanus peraccuratus, Procamallanus* sp., *Spirocamallanus hilarii, Spirocamallanus inopinatus, Spirocamallanus neocaballeroi, Spirocamallanus* sp., *Serpinema trispinosum*
*Astyanax jacuhiensis* (Cope, 1824)	*Spirocamallanus hilarii*
*Astyanax lacustris* (Lütken, 1874)	*Spirocamallanus inopinatus*
*Astyanax* sp.	*Procamallanus* sp., *Spirocamallanus inopinatus*
*Brachychalcinus copei* (Steindachner, 1882)	*Spirocamallanus inopinatus, Spirocamallanus* sp.
*Ctenobrycon* sp.	*Spirocamallanus inopinatus*
*Hyphessobrycon amapaensis* Zarske and Géry, 1998	*Camallanus* sp.
*Hyphessobrycon takasei* Géry, 1964	*Spirocamallanus inopinatus, Camallanus* sp.
*Moenkhausia costae* (Steindachner, 1907)	*Denticamallanus saofranciscensis, Procamallanus* sp., *Spirocamallanus neocaballeroi*
*Moenkhausia intermedia* Eigenmann, 1908	*Denticamallanus saofranciscensis, Spirocamallanus hilarii, Spirocamallanus inopinatus, Spirocamallanus* sp., *Serpinema trispinosum*
*Oligosarcus macrolepis* (Steindachner, 1877)	*Spirocamallanus hilarii*
*Paracheirodon axelrodi* (Schultz, 1956)	*Procamallanus* sp.
*Psalidodon fasciatus* (Cuvier, 1819)	*Denticamallanus saofranciscensis, Procamallanus* sp., *Spirocamallanus hilarii, Spirocamallanus inopinatus, Spirocamallanus* sp., *Serpinema trispinosum*
*Psalidodon* aff. *fasciatus*	*Spirocamallanus hilarii*
*Psalidodon parahybae* (Eigenmann, 1908)	*Spirocamallanus hilarii*
*Psalidodon schubarti* (Britski, 1964)	*Spirocamallanus hilarii, Spirocamallanus inopinatus*
**Family Acestrorhynchidae**	
*Acestrorhynchus falcatus* (Bloch, 1794)	*Spirocamallanus inopinatus*
*Acestrorhynchus falcirostris* (Cuvier, 1819)	*Spirocamallanus inopinatus*
*Acestrorhynchus heterolepis* (Cope, 1878)	*Spirocamallanus inopinatus*
*Acestrorhynchus lacustris* (Lütken, 1875)	*Denticamallanus saofranciscensis, Procamallanus* sp., *Spirocamallanus hilarii, Spirocamallanus inopinatus, Spirocamallanus neocaballeroi*
*Acestrorhynchus microlepis* (Jardine, 1841)	*Spirocamallanus hilarii*
**Family Achiridae**	
*Catathyridium jenynsii* (Günther, 1862)	*Spirocamallanus inopinatus*
**Family Anostomidae**	
Anostominae gen. sp.	*Denticamallanus iheringi*
*Anostomoides passionis* Santos and Zuanon, 2006	*Spirocamallanus inopinatus*
*Hypomasticus copelandii* (Steindachner, 1875)	*Denticamallanus iheringi, Procamallanus* sp., *Spirocamallanus inopinatus*
*Leporellus vittatus* (Valenciennes, 1850)	*Spirocamallanus inopinatus*
*Leporinus fasciatus* (Bloch, 1794)	*Denticamallanus iheringi, Spirocamallanus inopinatus*
*Leporinus friderici* (Bloch, 1794)	*Denticamallanus iheringi, Spirocamallanus amarali, Spirocamallanus inopinatus*
*Leporinus jamesi* Garman, 1929	*Procamallanus peraccuratus, Spirocamallanus inopinatus*
*Leporinus lacustris* Amaral Campos, 1945	*Spirocamallanus inopinatus*
*Leporinus moralesi* Fowler, 1942	*Spirocamallanus inopinatus*
*Leporinus octofasciatus* Steindachner, 1915	*Denticamallanus iheringi, Procamallanus* sp.
*Leporinus piau* Fowler, 1941	*Denticamallanus saofranciscensis, Spirocamallanus inopinatus, Spirocamallanus neocaballeroi*
*Leporinus* sp.	*Denticamallanus iheringi, Procamallanus* sp., *Spirocamallanus amarali, Spirocamallanus inopinatus*
*Leporinus striatus* Kner, 1858	*Procamallanus* sp., *Spirocamallanus inopinatus*
*Leporinus taeniatus* Lütken, 1875	*Spirocamallanus inopinatus, Serpinema trispinosum*
*Megaleporinus elongatus* (Valenciennes, 1850)	*Denticamallanus iheringi, Spirocamallanus amarali, Spirocamallanus inopinatus*
*Megaleporinus macrocephalus* (Garavello and Britski, 1988)	*Spirocamallanus inopinatus*
*Megaleporinus obtusidens* (Valenciennes, 1837)	*Denticamallanus iheringi, Spirocamallanus amarali, Spirocamallanus inopinatus*
*Megaleporinus reinhardti* (Lütken, 1875)	*Spirocamallanus inopinatus*
*Schizodon altoparanae* Garavello and Britski, 1990	*Procamallanus* sp.
*Schizodon borelli* (Boulenger, 1900)	*Denticamallanus iheringi, Spirocamallanus inopinatus*
*Schizodon fasciatus* Spix and Agassiz, 1829	*Denticamallanus iheringi, Spirocamallanus inopinatus*
*Schizodon knerii* (Steindachner, 1875)	*Spirocamallanus inopinatus*
*Schizodon nasutus* Kner, 1858	*Denticamallanus iheringi, Spirocamallanus inopinatus*
**Family Apteronotidae**	
*Sternarchella schotti* (Steindachner, 1868)	*Spirocamallanus inopinatus*
**Family Arapaimidae**	
*Arapaima gigas* (Schinz, 1822)	Camallanidae gen. sp., *Spirocamallanus inopinatus, Spirocamallanus* sp., *Camallanus tridentatus*
**Family Arhynchobatidae**	
*Atlantoraja castelnaui* (Miranda Ribeiro, 1907)	*Spirocamallanus pereirai, Spirocamallanus* sp.
**Family Auchenipteridae**	
*Ageneiosus inermis* (Linnaeus, 1766)	*Spirocamallanus inopinatus*
*Ageneiosus ucayalensis* Castelnau, 1855	*Denticamallanus rarus, Spirocamallaus belenensis*
*Ageneiosus* sp.	*Spirocamallanus inopinatus*
*Auchenipterichthys coracoideus* (Eigenmann and Allen, 1942)	*Spirocamallanus inopinatus*
*Auchenipterichthys thoracatus* (Kner, 1858)	*Spirocamallanus inopinatus*
*Auchenipterus ambyiacus* Fowler, 1915	*Spirocamallanus inopinatus*
*Auchenipterus brachyurus* (Cope, 1878)	*Spirocamallanus inopinatus*
*Auchenipterus nuchalis* (Spix and Agassiz, 1829)	*Spirocamallanus inopinatus, Spirocamallanus* sp.
*Auchenipterus osteomystax* (Miranda Ribeiro, 1918)	*Spirocamallanus* sp.
*Epapterus dispilurus* Cope, 1878	*Spirocamallanus inopinatus*
*Trachelyopterus galeatus* (Linnaeus, 1766)	*Procamallanus peraccuratus, Spirocamallanus inopinatus*
*Trachelyopterus striatulus* (Steindachner, 1877)	*Procamallanus peraccuratus*
**Family Batrachoididae**	
*Porichthys porosissimus* (Cuvier, 1829)	*Spirocamallanus* sp.
**Family Bryconidae**	
*Brycon amazonicus* (Spix and Agassiz, 1829)	*Spirocamallanus inopinatus*
*Brycon cephalus* (Günther, 1869)	*Spirocamallanus inopinatus*
*Brycon falcatus* Müller and Troschel, 1844	*Spirocamallanus inopinatus*
*Brycon hilarii* (Valenciennes, 1850)	Camallanidae gen. sp., *Spirocamallanus inopinatus*
*Brycon insignis* Steindachner, 1877	*Procamallanus* sp.
*Brycon orbignyanus* (Valenciennes, 1850)	*Spirocamallanus inopinatus*
*Brycon* sp.	*Spirocamallanus inopinatus, Spirocamallanus* sp.
*Brycon pesu* Müller and Troschel, 1845	*Spirocamallanus* sp.
*Salminus brasiliensis* (Cuvier, 1816)	*Spirocamallanus inopinatus*
*Salminus hilarii* Valenciennes, 1850	*Denticamallanus iheringi, Procamallanus* sp., *Spirocamallanus hilarii, Spirocamallanus* sp.
**Family Callichthyidae**	
*Corydoras amapaensis* Nijssen, 1972	*Spirocamallanus inopinatus*
*Corydoras armatus* (Günther, 1868)	*Spirocamallanus* sp.
*Corydoras ephippifer* Nijssen, 1972	*Spirocamallanus inopinatus, Camallanus* sp.
*Corydoras melanistius* Regan, 1912	*Spirocamallanus inopinatus, Camallanus* sp.
*Corydoras multiradiatus* (Orcés V., 1960)	*Spirocamallanus pintoi*
*Corydoras paleatus* (Jenyns, 1842)	*Spirocamallanus pintoi*
*Corydoras spilurus* Norman, 1926	*Spirocamallanus inopinatus*
*Corydoras trilineatus* Cope, 1872	*Spirocamallanus pintoi*
**Family Chalceidae**	
*Chalceus epakros* Zanata and Toledo-Piza, 2004	*Spirocamallanus inopinatus*
**Family Characidae**	
Characidae gen. sp.	*Denticamallanus iheringi, Procamallanus* sp.
*Charax gibbosus* (Linnaeus, 1758)	*Camallanus* sp.
*Charax pauciradiatus* (Günther, 1864)	*Spirocamallanus inopinatus, Spirocamallanus* sp.
*Cheirodon jaguaribensis* Fowler, 1941	*Spirocamallanus inopinatus, Spirocamallanus* sp., *Serpinema trispinosum*
*Cynopotamus kincaidi* (Schultz, 1950)	*Spirocamallanus* sp.
*Galeocharax humeralis* (Valenciennes, 1834)	*Spirocamallanus inopinatus*
*Roeboides affinis* (Günther, 1868)	*Spirocamallanus* sp.
*Roeboides myersii* Gill, 1870	*Spirocamallanus inopinatus*
*Roeboides* sp.	*Spirocamallanus inopinatus*
*Serrapinnus heterodon* (Eigenmann, 1915)	*Denticamallanus saofranciscensis, Spirocamallanus hilarii, Spirocamallanus inopinatus, Spirocamallanus* sp., *Serpinema trispinosum*
*Serrapinnus piaba* (Lutken, 1875)	*Spirocamallanus hilarii, Spirocamallanus* sp., *Serpinema trispinosum*
Tetragonopterinae gen. sp.	*Denticamallanus iheringi, Procamallanus* sp.
*Tetragonopterus argenteus* Cuvier, 1816	*Spirocamallanus inopinatus*
*Tetragonopterus chalceus* Spix and Agassiz, 1829	*Denticamallanus saofranciscensis, Spirocamallanus inopinatus, Spirocamallanus* sp.
*Tetragonopterus* sp.	*Denticamallanus iheringi*
**Family Cichlidae**	
*Astronotus ocellatus* (Agassiz, 1831)	*Denticamallanuss spiculastriatus, Spirocamallanus inopinatus, Camallanus* sp., *Serpinema trispinosum*
*Australoheros facetus* (Jenyns, 1842)	*Procamallanus peraccuratus, Procamallanus* sp.
*Biotodoma cupido* (Heckel, 1840)	*Procamallanus peraccuratus, Spirocamallanus inopinatus, Spirocamallanus pintoi, Spirocamallanus* sp.
*Bujurquina cordemadi* Kullander, 1986	*Spirocamallanus pimelodus*
*Cichla kelberi* Kullander and Ferreira, 2006	*Spirocamallanus inopinatus*
*Cichla monoculus* Agassiz, 1831	*Procamallanus peraccuratus, Spirocamallanus hilarii, Spirocamallanus inopinatus, Spirocamallanus neocaballeroi, Serpinema trispinosum*
*Cichla nigromaculata* Jardine and Schomburgk, 1843	*Spirocamallanus inopinatus*
*Cichla ocellaris* Bloch and Schneider, 1801	*Procamallanus peraccuratus*
*Cichla piquiti* Kullander and Ferreira, 2006	*Procamallanus peraccuratus*
*Cichla* sp.	*Spirocamallanus inopinatus*
*Cichlasoma amazonarum* Kullander, 1983	*Procamallanus* sp.
*Cichlasoma bimaculatum* (Linnaeus, 1758)	*Spirocamallanus inopinatus*
*Cichlasoma orientale* Kullander, 1983	*Serpinema trispinosum*
*Crenicichla brasiliensis* (Bloch, 1792)	*Denticamallanus krameri, Spirocamallanus hilarii, Spirocamallanus* sp., *Serpinema trispinosum*
*Crenicichla haroldoi* Luengo and Britski, 1974	*Spirocamallanus inopinatus*
*Crenicichla lepidota* Heckel, 1840	*Procamallanus peraccuratus*
*Crenicichla niederleini* (Holmberg, 1891)	*Procamallanus peraccuratus*
*Crenicichla* sp.	*Procamallanus peraccuratus, Spirocamallanus inopinatus*
*Geophagus altifrons* Heckel, 1840	*Spirocamallanus inopinatus*
*Geophagus brasiliensis* (Quoy and Gaimard, 1824)	*Procamallanus peraccuratus, Spirocamallanus hilarii*
*Geophagus iporangensis* Haseman, 1911	*Procamallanus* sp.
*Heros severus* Heckel, 1840	*Spirocamallanus inopinatus, Spirocamallanus pimelodus*
*Laetacara flavilabris* (Cope, 1870)	*Procamallanus peraccuratus*
*Mesonauta festivus* (Heckel, 1840)	*Spirocamallanus inopinatus*
*Mesonauta* sp.	*Procamallanus* sp.
*Oreochromis niloticus* (Linnaeus, 1758)	*Denticamallanus saofranciscensis*
*Satanoperca jurupari* (Heckel, 1840)	*Denticamallanus rarus, Spirocamallanus inopinatus, Spirocamallanus* sp.
**Family Curimatidae**	
*Curimatella dorsalis* (Eigenmann and Eigenmann, 1889)	*Spirocamallanus inopinatus*
*Curimatella* sp.	*Spirocamallanus inopinatus*
*Potamorhina altamazonica* (Cope, 1878)	*Procamallanus peraccuratus, Spirocamallanus inopinatus*
*Steindachnerina bimaculata* (Steindachner, 1876)	*Spirocamallanus inopinatus*
*Steindachnerina elegans* (Steindachner, 1875)	*Spirocamallanus hilarii*
**Family Cyclopsettidae**	
*Citharichthys macrops* Dresel, 1885	*Spirocamallanus halitrophus*
*Syacium papillosum* (Linnaeus, 1758)	*Spirocamallanus halitrophus*
**Family Cynodontidae**	
*Cynodon gibbus* (Agassiz, 1829)	*Spirocamallanus inopinatus*
*Rhaphiodon vulpinus* Spix and Agassiz, 1829	*Spirocamallanus inopinatus, Spirocamallanus* sp.
**Family Cynoglossidae**	
*Symphurus tessellatus* (Quoy and Gaimard, 1824)	*Spirocamallanus* sp.
**Family Dactylopteridae**	
*Dactylopterus volitans* (Linnaeus, 1758)	*Spirocamallanus macaensis*
**Family Doradidae**	
Doradidae gen. sp.	*Procamallanus* sp.
*Amblydoras affinis* (Kner, 1855)	*Spirocamallanus inopinatus*
*Nemadoras humeralis* (Kner, 1855)	*Spirocamallanus inopinatus, Spirocamallanus pimelodus*
*Ossancora asterophysa* Birindelli and Sabaj Pérez, 2011	*Spirocamallanus inopinatus*
*Platydoras costatus* (Linnaeus, 1758)	*Spirocamallanus inopinatus*
*Pterodoras granulosus* (Valenciennes, 1821)	*Spirocamallanus inopinatus, Paracamallanus amazonensis*
*Rhinodoras dorbignyi* (Kner, 1855)	*Denticamallanus rarus, Procamallanus* sp.
*Trachydoras paraguayensis* (Eigenmann and Ward, 1907)	*Procamallanus* sp., *Spirocamallanus inopinatus*
**Family Eleotridae**	
*Eleotris pisonis* (Gmelin, 1789)	*Spirocamallanus inopinatus*
**Family Ephippidae**	
*Chaetodipterus faber* (Broussonet, 1782)	*Spirocamallanus macaensis, Spirocamallanus* sp.
**Family Erythrinidae**	
*Erythrinus erythrinus* (Bloch and Schneider, 1801)	*Spirocamallanus inopinatus*
*Hoplerythrinus unitaeniatus* (Spix and Agassiz, 1829)	*Denticamallanus krameri, Spirocamallanus inopinatus*
*Hoplias lacerdae* Miranda Ribeiro, 1908	*Spirocamallanus hilarii*
*Hoplias malabaricus* (Bloch, 1794)	*Denticamallanus iheringi, Procamallanus peraccuratus, Procamallanus* sp., *Spirocamallanus hilarii, Spirocamallanus inopinatus, Spirocamallanus paraensis*
*Hoplias* aff. *malabaricus*	*Denticamallanus iheringi, Procamallanus peraccuratus, Spirocamallanus amarali, Spirocamallanus hilarii, Spirocamallanus inopinatus*
*Hoplias* sp.	*Denticamallanus iheringi*
**Family Gasteropelecidae**	
*Thoracocharax stellatus* (Kner, 1858)	*Spirocamallanus inopinatus*
**Family Gempylidae**	
*Thyrsitops lepidopoides* (Cuvier, 1832)	*Spirocamallanus macaensis*
**Family Gymnotidae**	
*Gymnotus carapo* Linnaeus, 1758	*Procamallanus peraccuratus*
**Family Hemiodontidae**	
*Anodus elongatus* Agassiz, 1829	*Spirocamallanus* sp.
*Anodus orinocensis* (Steindachner, 1887)	*Spirocamallanus inopinatus*
*Bivibranchia fowleri* (Steindachner, 1908)	*Spirocamallanus* sp.
*Bivibranchia notata* Vari and Goulding, 1985	*Spirocamallanus* sp.
*Bivibranchia velox* (Eigenmann and Myers, 1927)	*Spirocamallanus* sp.
*Hemiodus microlepis* Kner, 1858	*Spirocamallanus inopinatus, Spirocamallanus* sp.
*Hemiodus unimaculatus* (Bloch, 1794)	*Spirocamallanus inopinatus, Spirocamallanus* sp.
**Family Heptapteridae**	
*Pimelodella lateristriga* (Lichtenstein, 1823)	*Denticamallanus rarus, Spirocamallanus pimelodus*
*Rhamdia quelen* (Quoy and Gaimard, 1824)	*Spirocamallanus hilarii*
**Family Iguanodectidae**	
*Bryconops alburnoides* Kner, 1858	*Denticamallanus dentatus, Procamallanus* sp.
*Bryconops* cf. *affinis*	*Denticamallanus krameri*
*Bryconops caudomaculatus* (Günther, 1864)	*Spirocamallanus inopinatus*
*Bryconops melanurus* (Bloch, 1794)	*Spirocamallanus inopinatus*
*Iguanodectes spilurus* (Günther, 1864)	*Procamallanus* sp.
**Family Loricariidae**	
*Aphanotorulus emarginatus* (Valenciennes, 1840)	*Spirocamallanus inopinatus*
*Hypostomus albopunctatus* (Regan, 1908)	*Denticamallanus annipetterae*
*Hypostomus commersoni* Valenciennes, 1836	*Spirocamallanus hilarii*
*Hypostomus regani* (Ihering, 1905)	*Denticamallanus annipetterae*
*Hypostomus* sp.	*Denticamallanus annipetterae*
*Loricariichthys derbyi* Fowler, 1915	*Spirocamallanus* sp.
*Megalancistrus parananus* (Peters, 1881)	*Denticamallanus annipetterae*
*Proloricaria prolixa* (Isbrücker and Nijssen, 1978)	*Spirocamallanus inopinatus, Spirocamallanus rebecae*
**Family Lutjanidae**	
*Lutjanus synagris* (Linnaeus, 1758)	*Spirocamallanus* sp.
**Family Mochokidae**	
*Synodontis clarias* (Linnaeus, 1758)	*Denticamallanus rarus, Procamallanus* sp., *Spirocamallanus pimelodus*
**Family Mullidae**	
*Mullus argentinae* Hubbs and Marini, 1933	*Spirocamallanus halitrophus, Spirocamallanus pereirai*
**Family Ophidiidae**	
*Genypterus brasiliensis* Regan, 1903	*Procamallanus* sp.
**Family Osphronemidae**	
*Betta splendens* Regan, 1910	*Camallanus cotti*
**Family Osteoglossidae**	
*Osteoglossum bicirrhosum* (Cuvier, 1829)	*Camallanus acaudatus*
**Family Paralichthyidae**	
*Paralichthys isosceles* Jordan, 1891	*Spirocamallanus halitrophus*
*Xystreurys rasile* (Jordan, 1891)	*Spirocamallanus halitrophus*
**Family Percophidae**	
*Percophis brasiliensis* Quoy and Gaimard, 1825	*Procamallanus* sp.
**Family Phycidae**	
*Urophycis brasiliensis* (Kaup, 1858)	*Spirocamallanus halitrophus, Spirocamallanus macaensis*
*Urophycis* sp.	*Spirocamallanus* sp.
**Family Pimelodidae**	
*Bergiaria westermanni* (Lütken, 1874)	*Spirocamallanus freitasi*
*Calophysus macropterus* (Lichtenstein, 1819)	*Procamallanus* sp., *Spirocamallanus inopinatus*
*Cheirocerus eques* Eigenmann, 1917	*Spirocamallanus inopinatus*
*Conorynchus conirostris* (Valenciennes, 1840)	*Procamallanus* sp., *Spirocamallanus* sp.
*Hypophthalmus edentatus* Spix and Agassiz, 1829	*Paracamallanus amazonensis*
*Iheringichthys labrosus* (Lütken, 1874)	*Spirocamallanus pimelodus*
*Pimelodina flavipinnis* Steindachner, 1876	*Spirocamallanus inopinatus*
*Pimelodus albicans* (Valenciennes, 1840)	*Denticamallanus rarus*
*Pimelodus blochii* Valenciennes, 1840	*Denticamallanus rarus, Spirocamallanus inopinatus, Spirocamallanus pimelodus*
*Pimelodus maculatus* Lacepède, 1803	*Denticamallanus rarus, Procamallanus* sp., *Spirocamallanus freitasi, Spirocamallanus pimelodus*
*Pimelodus ornatus* Kner, 1858	*Spirocamallanus inopinatus*
*Pimelodus ortmanni* Haseman, 1911	*Procamallanus peraccuratus, Spirocamallanus pimelodus*
*Pimelodus pictus* Steindachner, 1876	*Spirocamallanus inopinatus*
*Pimelodus pohli* Ribeiro and Lucena, 2006	*Spirocamallanus freitasi, Spirocamallanus pimelodus, Spirocamallanus* sp.
*Pimelodus* sp.	Camallanidae gen. sp., *Spirocamallanus freitasi, Spirocamallanus inopinatus*
*Propimelodus caesius* Parisi, Lundberg and DoNascimiento, 2006	*Spirocamallanus inopinatus*
*Propimelodus eigenmanni* (Van der Stigchel, 1946)	*Spirocamallanus delirae*
*Pseudoplatystoma corruscans* (Spix and Agassiz, 1829)	*Spirocamallanus* sp.
*Pseudoplatystoma fasciatum* (Linnaeus, 1766)	*Camallanus* sp.
*Sorubim lima* (Bloch and Schneider, 1801)	*Spirocamallanus inopinatus*
*Zungaro zungaro* (Humboldt, 1821)	*Denticamallanus iheringi*
**Family Pinguipedidae**	
*Pseudopercis numida* Miranda Ribeiro, 1903	*Procamallanus* sp.
*Pseudopercis semifasciata* (Cuvier, 1829)	*Procamallanus* sp.
**Family Poeciliidae**	
*Poecilia reticulata* Peters, 1859	*Camallanus cotti*
*Poecilia vivipara* Bloch and Schneider, 1801	*Spirocamallanus neocaballeroi, Serpinema trispinosum*
*Xiphophorus maculatus* (Günther, 1866)	*Camallanus cotti*
**Family Potamotrygonidae**	
*Potamotrygon motoro* (Müller and Henle, 1841)	*Procamallanus peraccuratus, Spirocamallanus inopinatus*
**Family Priacanthidae**	
*Priacanthus arenatus* Cuvier, 1829	*Oncophora melanocephala*
**Family Prochilodontidae**	
*Prochilodus brevis* Steindachner, 1875	*Spirocamallanus hilarii*
*Prochilodus lineatus* (Valenciennes, 1837)	*Procamallanus* sp., *Spirocamallanus inopinatus, Spirocamallanus* sp.
*Prochilodus nigricans* Spix and Agassiz, 1829	*Spirocamallanus inopinatus*
*Semaprochilodus insignis* (Jardine, 1841)	*Spirocamallanus inopinatus*
**Family Sciaenidae**	
*Menticirrhus americanus* (Linnaeus, 1758)	*Procamallanus* sp., *Spirocamallanus macaensis*
*Micropogonias furnieri* (Desmarest, 1823)	*Spirocamallanus pereirai*
*Micropogonias undulatus* (Linnaeus, 1766)	*Spirocamallanus macaensis, Spirocamallanus* sp.
*Nebris microps* Cuvier, 1830	*Spirocamallanus macaensis*
*Paralonchurus brasiliensis* (Steindachner, 1875)	*Spirocamallanus macaensis, Spirocamallanus pereirai, Spirocamallanus* sp.
*Plagioscion auratus* (Castelnau, 1855)	*Spirocamallanus macaensis, Spirocamallanus* sp.
*Plagioscion squamosissimus* (Heckel, 1840)	*Paracamallanus amazonensis*
*Stellifer brasiliensis* (Schultz, 1945)	*Spirocamallanus macaensis*
**Family Scombridae**	
*Auxis thazard* (Lacepède, 1800)	*Oncophora melanocephala*
*Sarda sarda* (Bloch, 1793)	*Procamallanus* sp., *Oncophora melanocephala*
*Thunnus albacares* (Bonnaterre, 1788)	*Oncophora melanocephala*
*Thunnus atlanticus* (Lesson, 1831)	*Oncophora melanocephala*
*Thunnus thynnus* (Linnaeus, 1758)	*Oncophora melanocephala*
**Family Serrasalmidae**	
*Colossoma macropomum* (Cuvier, 1816)	*Procamallanus* sp., *Spirocamallanus inopinatus, Spirocamallanus* sp.
*Colossoma macropomum* x *Piaractus brachypomus* (hybrid fish)	*Spirocamallanus inopinatus*
*Metynnis hypsauchen* (Müller and Troschel, 1844)	*Spirocamallanus inopinatus*
*Metynnis lippincottianus* (Cope, 1870)	*Spirocamallanus inopinatus*
*Metynnis luna* Cope, 1878	*Spirocamallanus inopinatus*
*Myloplus arnoldi* Ahl, 1936	*Spirocamallanus inopinatus*
*Myloplus asterias* (Müller and Troschel, 1844)	*Spirocamallanus inopinatus*
*Myloplus rubripinnis* (Müller and Troschel, 1844)	*Spirocamallanus inopinatus*
*Mylossoma duriventre* (Cuvier, 1818)	*Spirocamallanus inopinatus*
*Pygocentrus nattereri* Kner, 1858	Camallanidae gen. sp., *Procamallanus peraccuratus, Procamallanus* sp., *Spirocamallanus inopinatus*
*Pygocentrus piraya* (Cuvier, 1819)	*Spirocamallanus inopinatus*
*Serrasalmus altispinis* Merckx, Jégu and Santos, 2000	*Spirocamallanus inopinatus*
*Serrasalmus brandtii* Lütken, 1875	*Spirocamallanus inopinatus*
*Serrasalmus eigenmanni* Norman, 1929	*Spirocamallanus inopinatus*
*Serrasalmus maculatus* Kner, 1858	*Spirocamallanus inopinatus, Spirocamallanus pimelodus*
*Serrasalmus marginatus* Valenciennes, 1837	*Spirocamallanus inopinatus*
*Serrasalmus rhombeus* (Linnaeus, 1766)	*Spirocamallanus inopinatus*
*Serrasalmus spilopleura* Kner, 1858	*Spirocamallanus inopinatus*
*Serrasalmus* sp.	*Denticamallanus rarus*
**Family Soleidae**	
*Solea senegalensis* Kaup, 1858	*Spirocamallanus pimelodus*
**Family Trichiuridae**	
*Trichiurus lepturus* Linnaeus, 1758	*Procamallanus* sp.
**Family Trichomycteridae**	
*Trichomycterus brasiliensis* Lütken, 1874	*Denticamallanus chimuensis*
*Trichomycterus punctulatus* Valenciennes, 1846	*Spirocamallanus hilarii*
**Family Triglidae**	
*Prionotus punctatus* (Bloch, 1793)	*Procamallanus* sp.
**Family Triportheidae**	
*Triportheus angulatus* (Spix and Agassiz, 1829)	*Denticamallanus saofranciscensis, Spirocamallanus inopinatus, Spirocamallanus rebecae*
*Triportheus auritus* (Valenciennes, 1850)	*Spirocamallanus inopinatus*
*Triportheus curtus* (Garman, 1890)	*Spirocamallanus inopinatus*
*Triportheus elongatus* (Günther, 1864)	*Spirocamallanus inopinatus*
*Triportheus guentheri* (Garman, 1890)	*Denticamallanus saofranciscensis, Procamallanus* sp., *Spirocamallanus* sp.
*Triportheus nematurus* (Kner, 1858)	*Procamallanus* sp., *Spirocamallanus* sp.
*Triportheus rotundatus* (Jardine, 1841)	*Spirocamallanus inopinatus*
*Triportheus signatus* (Garman, 1890)	*Denticamallanus saofranciscensis, Spirocamallanus hilarii, Spirocamallanus inopinatus, Serpinema trispinosum*
*Triportheus trifurcatus* (Castelnau, 1855),	*Spirocamallanus inopinatus*
**Unidentified species**	
‘bagre’	*Spirocamallanus solani*
‘cará-cahimbo’	*Procamallanus* sp., *Spirocamallanus* sp.
‘cara-suja’	*Spirocamallanus macaensis*
‘catfish’	*Denticamallanus rarus*
‘ferreirinha’	*Procamallanus* sp.
‘jatuarama’	*Procamallanus* sp., *Spirocamallanus* sp.
‘jeju’	*Spirocamallanus paraensis*
‘lambari amarela’	*Spirocamallanus hilarii*
‘lambari de cauda vermelha’	*Spirocamallanus hilarii*
‘lambari de rabo amarelo’	*Spirocamallanus inopinatus*
‘linguadinho’	*Procamallanus* sp.
‘matrinchã’	*Spirocamallanus* sp.
‘peixe-cachorro’	*Procamallanus* sp., *Spirocamallanus* sp.
‘piau’	*Spirocamallanus inopinatus*
‘tabarana’	*Procamallanus* sp.
‘taguara’ or ‘cachimboré’	*Denticamallanus iheringi*
‘taguara’ or ‘chimboré’	*Procamallanus* sp.
**Reptilia**	
**Chelonians**	
**Family Chelidae**	
*Acanthochelys spixii* (Duméril and Bibron, 1835)	*Camallanus* sp.
*Hydromedusa tectifera* Cope, 1870	*Spirocamallanus* sp., *Camallanus* sp., *Serpinema emydidium*
*Mesoclemmys nasuta* (Schweigger, 1812)	*Serpinema monospiculatum*
*Mesoclemmys tuberculata* (Luederwaldt, 1926)	*Serpinema amazonicum, Serpinema monospiculatum*
*Phrynops geoffroanus* (Schweigger, 1812)	*Camallanus* sp., *Serpinema amazonicum, Serpinema monospiculatum*
*Phrynops hilarii* (Duméril and Bibron, 1835)	*Camallanus* sp.
**Family Emydidae**	
*Trachemys dorbigni* (Duméril and Bibron, 1835)	*Camallanus* sp., *Serpinema emydidium*
**Family Kinosternidae**	
*Kinosternon scorpioides* (Linnaeus, 1766)	*Serpinema magathi, Serpinema pelliculatum*
**Family Podocnemididae**	
*Podocnemis expansa* (Schweigger, 1812)	*Serpinema amazonicum, Serpinema microcephalus*
*Podocnemis unifilis* Troschel, 1848	*Serpinema microcephalus*
**Serpentes**	
**Family Viperidae**	
*Bothrops atrox* (Linnaeus, 1758)	*Camallanus* sp.

**Figure 6.**
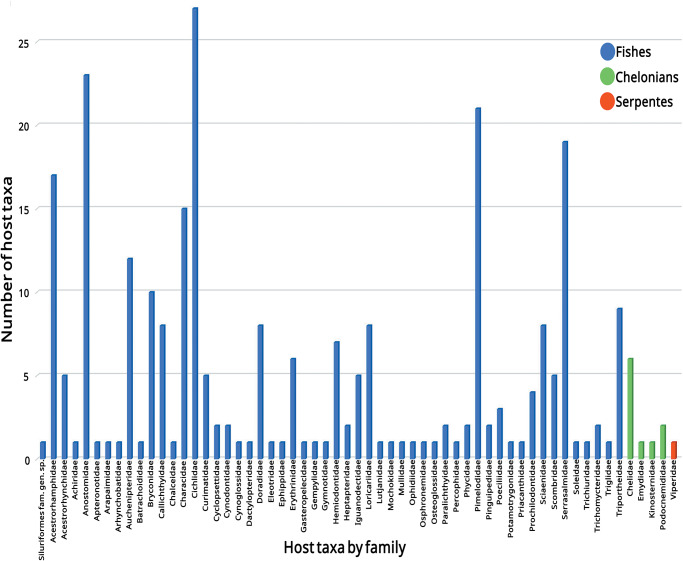
Number of host taxa of camallanids by the host families in Brazil. Figure 6 long description.

Among the host families, the most frequent were Cichlidae (27 species), followed by Anostomidae (23 taxa) and Pimelodidae (21 taxa), from fish hosts. Among the chelonians, the family Chelidae has the highest number of species parasitized by camallanids, with 6 host species recorded to date in Brazil. Viperidae was the only snake family represented, with only 1 species recorded (Fig. 6).

The North and Southeast regions of Brazil exhibited the highest diversity of Camallanidae, with 38 and 37 taxa reported, respectively. Among the Brazilian states, Pará (North) and São Paulo (Southeast) recorded the greatest number of taxa, with 15 and 14 species, respectively. Across Brazilian biomes, the Amazon exhibited the highest diversity of camallanids, with 22 taxa, followed by the Atlantic Forest (21), Cerrado (13), Caatinga (11), Pampa (6) and Pantanal (1).

Regarding host habitats, most camallanid species are associated with freshwater hosts, totalling 32 taxa. Marine and freshwater/terrestrial environments each harbour 6 taxa, whereas strictly terrestrial hosts contain only a single camallanid taxon.

### Checklist of species of the family Camallanidae reported in Brazil

**Camallanidae gen. sp.**

**Hosts**: *Arapaima gigas* (Schinz, 1822), *Brycon hilarii* (Valenciennes, 1850), *Pimelodus* sp., *Pygocentrus nattereri* Kner, 1858.

**Host environments**: freshwater.

**Sites of infection**: intestine.

**Locality records**: Midwest: Mato Grosso; North: Acre; Southeast: São Paulo.

**Biomes**: Amazon, Atlantic Forest and Cerrado.

**References:** Travassos (1940); Travassos and Freitas (1942); Silva et al. (2016).

**Unconfirmed hosts:** *Astyanax bimaculatus* (Linnaeus, 1758), *Cyphocharax gilbert* (Quoy and Gaimard, 1824), *Moenkhausia doceana* (Steindachner, 1877) in Luque et al. (2011).

**Unconfirmed locality records:** Espírito Santo (Southeast) in Luque et al. (2011).

**Subfamily Procamallaninae Yeh, 1960**

**Genus *Denticamallanus* Moravec and Thatcher, 1997**

***Denticamallanus dentatus* (Moravec and Thatcher, 1997)**

**Synonyms:** *Procamallanus* (*Denticamallanus*) *dentatus* Moravec and Thatcher, 1997.

**Hosts:** *Bryconops alburnoides* Kner, 1858.

**Host environments:** freshwater.

**Sites of infection:** intestine.

**Locality records:** North: Amazonas.

**Biomes:** Amazon.

**Specimens deposited:** holotype, allotype and paratypes: INPA-NEM 007-010; paratypes HWML 39128; paratypes in the Institute of Parasitology, Academy of Sciences of the Czech Republic, České Budějovice, Czech Republic, N–678.

**References:** Moravec and Thatcher (1997).

**Remarks:** *Denticamallanus* was proposed as a subgenus of *Procamallanus* by Moravec and Thatcher (1997), presenting as the main characteristic the presence of conical tooth-like structures in the posterior region of the buccal capsule in males and spiral ridges in females. By analysing records of other camallanid species, we observed that different species also present tooth-like structures, sometimes with sexual dimorphism. Therefore, our study considered the diagnostic characteristic for *Denticamallanus* to be the presence of conical tooth-like structures at the basal ring or in the posterior region of the buccal capsule of at least 1 sex, with spiral ridges that may or may not be present. These distinctive features, combined with the analyses of Ailán-Choke and Pereira (2021) (see Discussion), further support the recognition of *Denticamallanus* as a valid genus rather than a subgenus.

***Denticamallanus annipetterae* (Kohn and Fernandes, 1988)**

**Synonyms:** *Procamallanus annipetterae* Kohn and Fernandes, 1988; *Procamallanus* (*Procamallanus*) *annipetterae* Kohn and Fernandes, 1988; *Procamallanus petterae* Kohn and Fernandes, 1988.

**Hosts:** *Hypostomus albopunctatus* (Regan, 1908) (=*Plecostomus albopunctatus* Regan, 1908), *Hypostomus regani* (Ihering, 1905), *Hypostomus* sp., *Megalancistrus parananus* (Peters, 1881).

**Host environments:** freshwater.

**Sites of infection:** intestine.

**Locality records:** South: Paraná.

**Biomes:** Atlantic Forest.

**Specimens deposited:** holotype: CHIOC 32430a, allotype: CHIOC 32430b.

**References:** Kohn and Fernandes (1988a); Kohn et al. (2011).

**Unconfirmed hosts:** *Hypostomus ternetzi* (Boulenger, 1895) in Lehun et al. (2020).

**Remarks:** Two different species of *Procamallanus* were proposed with the same name: *Procamallanus* (*Procamallanus*) *patterae* Moravec and Sey, 1988, described in Vietnam, and *Procamallanus petterae* Kohn and Fernandes, 1988, described in Brazil. Following the International Code of Zoological Nomenclature, a new name for *Pr. petterae* Kohn and Fernandes, 1988, from Brazil, was proposed: *Procamallanus annipetterae* Kohn and Fernandes, 1988 (Kohn and Fernandes, 1988b).

*Denticamallanus annipetterae* (=*Procamallanus annipetterae*) was described with 5 tooth-like structures at the base of the buccal capsule and the absence of spiral ridges in both sexes. Additionally, we evaluated the allotype of the species (code CHIOC 32430b) and confirmed the characteristics of *Denticamallanus*. Therefore, we reallocated the species to the genus *Denticamallanus*, based on the new diagnostic criteria proposed (see key to genera of Procamallaninae).

***Denticamallanus chimuensis* (Freitas and Ibáñez, 1968)**

**Synonyms:** *Spirocamallanus chimuensis* Freitas and Ibáñez, 1968; *Procamallanus* (*Spirocamallanus*) *chimuensis* (Freitas and Ibáñez, 1968); *Procamallanus* (*Spirocamallanus*) *pexatus* Pinto, Fabio, Noronha and Rolas, 1976; *Procamallanus pexatus* Pinto, Fabio, Noronha and Rolas, 1976; *Spirocamallanus pexatus* (Pinto, Fabio, Noronha and Rolas, 1976).

**Hosts:** *Trichomycterus brasiliensis* Lütken, 1874 (=*Pygidium brasiliensis* Eigenmann and Eigenmann, 1889).

**Host environments:** freshwater.

**Sites of infection:** intestine.

**Locality records:** Southeast: Espírito Santo.

**Biomes:** Atlantic Forest.

**Specimens deposited:** vouchers CHIOC 14228, 14233, 31086a-d, 31087, 31088a, 31088b, 31090a, 31089a-b, 31090b-c.

**References:** Pinto et al. (1976).

**Remarks:** This record refers to the study by Pinto et al. (1976), which described the species *Procamallanus* (*Spirocamallanus*) *pexatus* Pinto, Fabio, Noronha and Rolas, 1976. However, the species was subsequently synonymized with *D. chimuensis* (=*Sp. chimuensis*) by Moravec et al. (2004). In both studies, the authors describe the species as having spiral ridges in males and females. Moravec et al. (2004) also report the presence of 3 tooth-like structures in the buccal capsule of females. Despite not presenting in the description, the illustrations provided by Pinto et al. (1976) also show the presence of tooth-like structures in the buccal capsule of females. Therefore, we reallocated the species to the genus *Denticamallanus*.

***Denticamallanus iheringi* (Travassos, Artigas and Pereira, 1928)**

**Synonyms:** *Procamallanus iheringi* Travassos, Artigas and Pereira, 1928; *Spirocamallanus iheringi* (Travassos, Artigas and Pereira, 1928); *Procamallanus* (*Spirocamallanus*) *iheringi* (Travassos, Artigas and Pereira, 1928).

**Hosts:** *Hoplias malabaricus* (Bloch, 1794), *Hoplias* aff*. malabaricus, Hoplias* sp., *Hypomasticus copelandii* (Steindachner, 1875) (=*Leporinus copelandii* Steindachner, 1875), *Leporinus fasciatus* (Bloch, 1794), *Leporinus friderici* (Bloch, 1794), *Leporinus octofasciatus* Steindachner, 1915, *Leporinus* sp., *Megaleporinus elongatus* (Valenciennes, 1850) (=*Leporinus elongatus* Valenciennes, 1850), *Megaleporinus obtusidens* (Valenciennes, 1837) (=*Leporinus obtusidens* (Valenciennes, 1837))*, Salminus hilarii* Valenciennes, 1850, *Schizodon borelli* (Boulenger, 1900), *Schizodon fasciatus* Spix and Agassiz, 1829, *Schizodon nasutus* Kner, 1858, *Tetragonopterus* sp., *Zungaro zungaro* (Humboldt, 1821) (=*Pseudopimelodus zungaro* (Humboldt, 1821)), Anostominae gen. sp., Tetragonopterinae gen. sp., Characidae gen. sp., ‘taguara’ or ‘cachimboré’ (local names).

**Host environments:** freshwater.

**Sites of infection:** intestine, pyloric cecum, pyloric diverticulum.

**Locality records:** South: Paraná; Southeast: Minas Gerais, São Paulo.

**Biomes:** Atlantic Forest and Cerrado.

**Specimens deposited:** type-material CHIOC 5875, vouchers CHIOC 14739, 16473-16478, 16480,16481, 16483-16488, 16490-16493, 16495, 16497-16505, 16508, 16511, 16514, 28837, 31063a-d, 31070a-b, 31319, 31320, 31321.

**References:** Travassos et al. (1928); Travassos and Kohn (1965); Kohn and Fernandes (1987); Pinto et al. (1975); Pinto and Noronha (1976); Moravec et al. (1993); Machado et al. (1995); Machado et al. (1996); Feltran et al. (2004); Pavanelli et al. (2004); Guidelli et al. (2006); Takemoto et al. (2009); Kohn et al. (2011); Corrêa et al. (2020).

**Unconfirmed hosts:** *Psalidodon fasciatus* (Cuvier, 1819) (=*Astyanax fasciatus* (Cuvier, 1819)) in Luque et al. (2011); *Megaleporinus piavussu* (Britski, Birindelli and Garavello, 2012) in Lehun et al. (2020).

**Unconfirmed locality records:** Espírito Santo (Southeast) in Luque et al. (2011).

**Remarks:** Pinto et al. (1975) redescribed *D. iheringi* (=*Sp. iherengi*) with illustrations that show the presence of tooth-like structures in the buccal capsule of females, although the authors did not report this in their description. Additionally, the authors indicate the codes 31070 a-b as the type material, but the type material registered and deposited in CHIOC is under code CHIOC 5857.

Moravec et al. (1993) also illustrate the presence of 6 tooth-like structures in the buccal capsule of females of *D. ihering* (=*Sp. iherengi*). Therefore, we reallocated the species to the genus *Denticamallanus*.

***Denticamallanus krameri* (Petter, 1974)**

**Synonyms:** *Spirocamallanus krameri* Petter, 1974; *Procamallanus* (*Spirocamallanus*) *krameri* (Petter, 1974).

**Hosts:** *Bryconops* cf. *affinis, Hoplerythrinus unitaeniatus* (Spix and Agassiz, 1829), *Crenicichla brasiliensis* (Bloch, 1792) (=*Saxatilia brasiliensis* (Bloch, 1792)).

**Host environments:** freshwater.

**Sites of infection:** middle intestine, pyloric cecum.

**Locality records:** North: Pará; Northeast: Maranhão.

**Biomes:** Amazon and Cerrado.

**Specimens deposited:** vouchers MPEG 0206, 0207, 0208, 0209, (Access number: 20190400001).

**References:** Pinheiro et al. (2020); Pinheiro et al. (2021); Cárdenas et al. (2024).

**Remarks:** *Denticamallanus krameri* (=*Sp. krameri*) was described by Petter (1974) into the genus *Spirocamallanus*, based on only 1 male and 1 female specimen. The species was described parasitizing *Hoplerythrinus uniateniatus* in French Guiana. Moravec et al. (1997) present the first redescription of the species from specimens collected from the type host from Venezuela. Both studies report the presence of tooth-like structures only in males.

Pinheiro et al. (2020) present a new redescription of *D. krameri* (=*Sp. krameri*), with the first record of this species in Brazil. The study includes scanning electron microscopy (SEM) analysis of the species but does not provide light microscopy (photomicrographs or line drawings), so there is no representation of the internal morphology of the buccal capsule. However, the authors report, in addition to spiral ridges in both sexes, 3 large teeth at the base of the basal ring in males, absent in females. Additionally, Cárdenas et al. (2024) also report spiral ridges in both sexes and the presence of 3 tooth-like structures at the base of the buccal capsule of males. Therefore, we reallocated the species to the genus *Denticamallanus*.

***Denticamallanus rarus* (Travassos, Artigas and Pereira, 1928)**

**Synonyms:** *Spirocamallanus rarus* Travassos, Artigas and Pereira, 1928; *Procamallanus rarus* (Travassos, Artigas and Pereira, 1928); *Procamallanus* (*Spirocamallanus*) *rarus* (Travassos, Artigas and Pereira, 1928).

**Hosts:** *Ageneiosus ucayalensis* Castelnau, 1855, *Pimelodella lateristriga* (Lichtenstein, 1823), *Pimelodus albicans* (Valenciennes, 1840), *Pimelodus blochii* Valenciennes, 1840, *Pimelodus maculatus* Lacepède, 1803, *Rhinodoras dorbignyi* (Kner, 1855), *Satanoperca jurupari* (Heckel, 1840), *Serrasalmus* sp., *Synodontis clarias* (Linnaeus, 1758) (=*Pimelodus clarias* Geoffroy Saint-Hilaire, 1809), ‘catfish’ (undetermined species).

**Host environments:** freshwater.

**Sites of infection:** intestine; small intestine.

**Locality records:** North: Acre, Pará; Southeast: São Paulo.

**Biomes:** Amazon, Atlantic Forest and Cerrado.

**Specimens deposited:** type material CHIOC 5930; vouchers CHIOC 6055, 26411, 26414, 31026-31029, 35717.

**References:** Travassos et al. (1928); Travassos and Kohn (1965); Pinto et al. (1974); Kohn and Fernandes (1987); Giese et al. (2009); Melo et al. (2011); Negreiros et al. (2018); Cavalcante et al. (2020).

**Unconfirmed hosts:** *Cichla piquiti* Kullander and Ferreira, 2006 and *Sorubim lima* (Bloch and Schneider, 1801) in Lehun et al. (2020).

**Remarks:** Moravec (1998) states that the characterization of the spicule of *D. rarus* (=*Sp. rarus*) in the study by Travassos etal. (1928) is inadequate and provides a more detailed description of this structure. Melo et al. (2011) presents a description of *D. rarus* (=*Sp. rarus*) that matches the description provided in Moravec (1998). Thus, we reallocated the species to the genus *Denticamallanus* based on the illustrations from the studies by Pinto et al. (1974), Moravec (1998) and Melo et al. (2011), which suggest the presence of 3 or 4 tooth-like structures in the buccal capsule of males.

***Denticamallanus saofranciscensis* (Moreira, Oliveira and Costa, 1994)**

**Synonyms:** *Spirocamallanus saofranciscensis* Moreira, Oliveira and Costa, 1994; *Procamallanus* (*Spirocamallanus*) *saofranciscencis* (Moreira, Oliveira and Costa, 1994).

**Hosts:** *Acestrorhynchus lacustris* (Lütken, 1875), *Astyanax bimaculatus, Leporinus piau* Fowler, 1941, *Moenkhausia costae* (Steindachner, 1907), *Moenkhausia intermedia* Eigenmann, 1908, *Oreochromis niloticus* (Linnaeus, 1758), *Psalidodon fasciatus* (=*Astyanax fasciatus*), *Serrapinnus heterodon* (Eigenmann, 1915), *Tetragonopterus chalceus* Spix and Agassiz, 1829, *Triportheus angulatus* (Spix and Agassiz, 1829), *Triportheus guentheri* (Garman, 1890), *Triportheus signatus* (Garman, 1890).

**Host environments:** freshwater.

**Sites of infection:** coelomic cavity, intestinal cecum, intestine, mesentery, pyloric cecum, stomach.

**Locality records:** Northeast: Bahia, Ceará, Paraíba, Rio Grande do Norte; Southeast: Minas Gerais, São Paulo.

**Biomes:** Caatinga and Cerrado.

**Specimens deposited:** vouchers CHIOC 35578, 37857, 37858; voucher CHIBB 6815.

**References:** Moreira et al. (1994); Abdallah et al. (2012); Albuquerque et al. (2016); Laurentino E Silva et al. (2017); Vieira-Menezes et al. (2017); Duarte et al. (2022a); Falkenberg et al. (2024); Sousa et al. (2025).

**Unconfirmed specimens deposited:** deposit codes CHIOC 39115, 39116 in Duarte et al. (2022a).

**Remarks:** Moreira et al. (1994) described *D. saofranciscensis* (=*Sp. saofranciscensis*) with 3 teeth at the base of the buccal capsule in males and females. Therefore, we reallocated the species to the genus *Denticamallanus*. The authors mention the deposit of the type material in CHIOC but do not provide the codes. Consulting the CHIOC online catalogue, none of the materials identified as ‘*Spirocamallanus saofranciscensis*’ refer to the material analysed by Moreira et al. (1994). Additionally, the deposit codes CHIOC 39115 and 39116 provided in the study by Duarte et al. (2022a) are not included in the CHIOC online catalogue.

We also analysed specimen vouchers of *D. saofranciscensis* (=*Sp. saofranciscensis*) (codes CHIOC 37857 and 37858) and confirmed the presence of tooth-like structures in both sexes.

***Denticamallanus spiculastriatus* (Pinheiro, Melo, Monks, Santos and Giese, 2018)**

**Synonyms:** *Procamallanus spiculastriatus* Pinheiro, Melo, Monks, Santos and Giese, 2018.

**Hosts:** *Astronotus ocellatus* (Agassiz, 1831).

**Host environments:** freshwater.

**Sites of infection:** intestine.

**Locality records:** North: Pará.

**Biomes:** Amazon.

**Specimens deposited:** holotype MPEG 195, allotype MPEG 196, paratypes MPEG 197–200.

**References:** Pinheiro et al. (2018); Pinheiro et al. (2019).

**Remarks:** Pinheiro et al. (2018) described *D. spiculastriatus* (=*Pr. spiculastriatus*) with the inner surface of the buccal capsule smooth, without ridges, in both sexes. However, in the figures and discussion of the article, the authors indicate the presence of 4 tooth-like structures at the basal ring in both sexes. Therefore, we reallocated the species to the genus *Denticamallanus*.

**Genus *Procamallanus* Baylis, 1923**

***Procamallanus peraccuratus* Pinto, Fabio, Noronha and Rolas, 1976**

**Synonyms:** *Procamallanus* (*Procamallanus*) *peraccuratus* Pinto, Fabio, Noronha and Rolas, 1976.

**Hosts:** *Astyanax bimaculatus, Australoheros facetus* (Jenyns, 1842) (=*Cichlasoma facetum* (Jenyns, 1842), *Cichlaurus facetus* (Jenyns, 1842)), *Biotodoma cupido* (Heckel, 1840), *Cichla monoculus* Agassiz, 1831, *Cichla ocellaris* Bloch and Schneider, 1801, *Cichla piquiti, Crenicichla lepidota* Heckel, 1840, *Crenicichla niederleinii* (Holmberg, 1891), *Crenicichla* sp., *Geophagus brasiliensis* (Quoy and Gaimard, 1824), *Gymnotus carapo* Linnaeus, 1758, *Hoplias malabaricus, Hoplias* aff*. malabaricus, Laetacara flavilabris* (Cope, 1870), *Leporinus jamesi* Garman, 1929, *Pimelodus ortmanni* Haseman, 1911, *Potamorhina altamazonica* (Cope, 1878), *Potamotrygon motoro* (Müller and Henle, 1841), *Pygocentrus nattereri, Trachelyopterus galeatus* (Linnaeus, 1766), *Trachelyopterus striatulus* (Steindachner, 1877).

**Host environments:** freshwater.

**Sites of infection:** intestine, mesentery, stomach.

**Locality records:** North: Acre; Amapá; Northeast: Paraíba; South: Paraná, Rio Grande do Sul; Southeast: Espírito Santo, Rio de Janeiro, São Paulo.

**Biomes:** Amazon, Atlantic Forest, Caatinga and Pampa.

**Specimens deposited:** holotype CHIOC 31084a, allotype 31084b, paratypes CHIOC 31078, 31079, 31080 a-b, 31081 a-b, 31082 a-b, 31083 a-d, 31084c, 31085, 16744-16749, 16751-16754, 16757-16776, 16853, 29446, 29472; vouchers CHIBB 4999, 5000, 5006, 5145.

**References:** Pinto et al. (1976); Kohn et al. (1988); Kohn et al. (1989); Welblen and Brandão (1992); Moravec et al. (1993); Pavanelli et al. (2004); Takemoto et al. (2009); Azevedo et al. (2010); Carvalho et al. (2010); Bellay et al. (2011); Kohn et al. (2011); Mesquita et al. (2011); Franceschini et al. (2013); Virgilio et al. (2022); Mota-Júnior et al. (2024); De Lima et al. (2025).

**Unconfirmed hosts:** *Schizodon nasutus* in Luque et al. (2011); *Hemisorubim platyrhynchos* (Valenciennes, 1840), *Hoplias* spp. and *Potamotrygon amandae* Loboda and Carvalho, 2013 in Lehun et al. (2020).

**Remarks:** *Procamallanus peraccuratus* was described from material previously deposited in CHIOC.

Mota-Júnior et al. (2024) report ‘*Spirocamallanus peraccuratus’* in *Crenicichla strigata* from Amapá State (North). However, this combination of names does not exist in literature. Based on the specific epithet ‘*peraccuratus*’ and its occurrence in Brazil, probably there was a mistake in the authors’ writing, and it refers to the species *Pr. peraccuratus*. This species is very well described and characterized as belonging to the genus *Procamallanus*, with no doubt so far regarding its taxonomic classification.

***Procamallanus* sp.**

**Hosts:** *Acestrorhynchus lacustris, Astyanax bimaculatus, Astyanax* sp., *Australoheros facetus, Brycon insignis* Steindachner, 1877, *Bryconops alburnoides, Calophysus macropterus* (Lichtenstein, 1819), *Cichlasoma amazonarum* Kullander, 1983, *Colossoma macropomum* (Cuvier, 1816), *Conorynchus conirostris* (Valenciennes, 1840) (=*Conostome conirostris* Valenciennes, 1840), *Genypterus brasiliensis* Regan, 1903, *Geophagus iporangensis* Haseman, 1911, *Hoplias malabaricus, Hypomasticus copelandii* (=*Leporinus copelandii*), *Iguanodectes spilurus* (Günther, 1864), *Leporinus octofasciatus, Leporinus striatus* Kner, 1858, *Leporinus* sp., *Menticirrhus americanus* (Linnaeus, 1758), *Mesonauta* sp., *Moenkhausia costae, Paracheirodon axelrodi* (Schultz, 1956), *Percophis brasiliensis* Quoy and Gaimard, 1825, *Pimelodus maculatus, Prionotus punctatus* (Bloch, 1793), *Prochilodus lineatus* (Valenciennes, 1837) (=*Prochilodus scrofa* Steindachner, 1881)*, Psalidodon fasciatus* (=*Astyanax fasciatus*), *Pseudopercis numida* Miranda Ribeiro, 1903, *Pseudopercis semifasciata* (Cuvier, 1829), *Pygocentrus nattereri, Rhinodoras dorbignyi, Salminus hilarii, Sarda sarda* (Bloch, 1793), *Schizodon altoparanae* Garavello and Britski, 1990, *Synodontis clarias* (=*Pimelodus clarias*), *Trachydoras paraguayensis* (Eigenmann and Ward, 1907), *Trichiurus lepturus* Linnaeus, 1758, *Triportheus nematurus* (Kner, 1858) (=*Chalcinus nematurus* Kner, 1858), *Triportheus guentheri,* Characidae gen. sp., Doradidae gen. sp., Tetragonopterinae gen. sp., Siluriformes fam. gen. sp., ‘jatuarama’, ‘cará-cahimbo’, ‘ferreirinha’, ‘linguadinho’, ‘peixe-cachorro’, ‘tabarana’, ‘taguara’ or ‘chimboré’ (local names).

**Host environments:** freshwater and marine.

**Sites of infection:** intestine, large intestine, mesentery, pyloric diverticulum, small intestine, stomach.

**Locality records:** Midwest: Mato Grosso; North: Amapá, Amazonas, Pará; Northeast: Maranhão, Paraíba; South: Paraná; Southeast: Espírito Santo, Minas Gerais; Rio de Janeiro, São Paulo.

**Biomes:** Amazon, Atlantic Forest and Caatinga.

**Specimens deposited:** CHIOC 8625, 8629, 8650, 8752, 8816, 8817, 8829, 12273, 12283, 14722, 14723, 14734, 14738, 14751, 14763, 16482, 16489, 16494, 16512, 16519, 16520, 16521, 16777, 16789, 16792, 16799, 16788, 16794, 16798, 26494, 28439, 28444, 28452, 28475, 28476, 28477, 28478, 28629, 30804a-b, 31313, 31314a-b, 31344, 31345, 31346a-b, 34653, 35350, 35461, 35577.

**References:** Travassos and Freitas (1940); Travassos and Freitas (1948); Travassos and Freitas (1964); Vicente and Santos (1973); Pinto et al. (1975); Pinto and Noronha (1976); Kohn et al. (1985); Kohn and Fernandes (1987); Kohn et al. (1988); Silva et al. (2000); Alves et al. (2002); Carvalho et al. (2003); Fischer et al. (2003); Pavanelli et al. (2004); Bicudo et al. (2005); Alves and Luque (2006); Fernandes et al. (2006); Santos et al. (2007); Luque et al. (2008); Takemoto et al. (2009); Tavares-Dias et al. (2009); Azevedo et al. (2010); Barros et al. (2010); Tavares-Dias et al. (2010); Benigno et al. (2012); Fujimoto et al. (2013); Bittencourt et al. (2014); Albuquerque et al. (2016); Vieira-Menezes et al. (2017); Cárdenas et al. (2022); Moraes et al. (2024); De Lima et al. (2025); Sousa et al. (2025).

**Unconfirmed hosts:** *Brevoortia aurea* (Spix and Agassiz, 1829)*, Hemiodus* sp., *Megaleporinus macrocephalus* (=*Leporinus macrocephalus* Garavello and Britski, 1988) (Garavello and Britski, 1988), *Pimelodus blochii, Serrasalmus marginatus* Valenciennes, 1837, and *Serrasalmus spilopleura* Kner, 1858 in Eiras et al. (2016).

**Remarks:** All the studies mentioned identified camallanids only at the genus level. However, many authors used, and still use, the subgeneric classification for the genus *Procamallanus*. Therefore, these specimens identified as ‘*Procamallanus* sp.’ may belong to other genera in the family, especially *Spirocamallanus*.

**Genus *Spirocamallanus* Olsen, 1952**

***Spirocamallanus amarali*** **(Vaz and Pereira, 1934)**

**Synonyms:** *Procamallanus amarali* Vaz and Pereira, 1934; *Procamallanus* (*Spirocamallanus*) *amarali* Vaz and Pereira, 1934.

**Hosts:** *Hoplias* aff. *malabaricus, Leporinus* sp., *Leporinus friderici, Megaleporinus elongatus* (=*Leporinus elongatus*), *Megaleporinus obtusidens* (=*Leporinus obtusidens*).

**Host environments:** freshwater.

**Sites of infection:** anterior intestine, cecum, intestine, middle intestine, pyloric diverticulum.

**Locality records:** South: Paraná; Southeast: São Paulo.

**Biomes:** Atlantic Forest and Cerrado.

**Specimens deposited:** CHIOC 16506, 31064a-r.

**References:** Vaz and Pereira (1934); Kohn and Fernandes (1987); Pinto et al. (1975); Guidelli et al. (2006); Takemoto et al. (2009); Corrêa et al. (2020).

**Unconfirmed hosts:** *Hypomasticus copelandii* (=*Leporinus copelandii*) in Luque et al. (2011); *Megaleporinus piavussu* in Lehun et al. (2020).

**Remarks:** Pinto et al. (1975) described the female of *Sp. amarali* for the first time and redescribed the male of the species.

***Spirocamallanus belenensis*** **(Giese, Santos and Lanfredi, 2009)**

**Synonyms:** *Procamallanus belenensis* Giese, Santos and Lanfredi, 2009; *Procamallanus* (*Spirocamallanus*) *belenensis* Giese, Santos and Lanfredi, 2009.

**Hosts:** *Ageneiosus ucayalensis*.

**Host environments:** freshwater.

**Sites of infection:** abdominal cavity, intestine.

**Locality records:** North: Amapá, Pará.

**Biomes:** Amazon.

**Specimens deposited:** type material CHIOC 35604a-c.

**References:** Giese et al. (2009); Ferreira and Tavares-Dias (2017).

**Remarks:** *Spirocamallanus belenensis* was described by Giese et al. (2009) into the genus *Procamallanus*, subgenus *Spirocamallanus*, due to the presence of spiral ridges in buccal capsule of both sexes. However, currently the genus status of *Spirocamallanus* is accepted (Ailán-Choke and Pereira, 2021). Therefore, we considered the species belonging to genus *Spirocamallanus*.

***Spirocamallanus caballeroi* Bashirullah, 1977**

**Synonyms:** *Spirocamallanus dessetae* Petter, Golvan and Tcheprakoff, 1977.

**Hosts**: *Astyanax altiparanae* Garutti and Britski, 2000.

**Host environments:** freshwater.

**Sites of infection:** not provided.

**Locality records:** South: Paraná.

**Biomes:** Atlantic Forest.

**References:** Pavanelli et al. (2004); Takemoto et al. (2009).

**Unconfirmed hosts:** *Astyanax lacustris* (Lütken, 1874) in Lehun et al. (2020).

***Spirocamallanus delirae* Ruffeil, Giese and Pinheiro, 2023**

**Hosts:** *Propimelodus eigenmanni* (Van der Stigchel, 1946).

**Host environments:** freshwater.

**Sites of infection:** intestine.

**Locality records:** North: Pará.

**Biomes:** Amazon.

**Specimens deposited:** holotype MPEG 0277, allotype MPEG 0278, paratypes MPEG 0279, 0280.

**References:** Ruffeil et al. (2023).

***Spirocamallanus freitasi* Moreira, Oliveira and Costa, 1991**

**Hosts:** *Bergiaria westermanni* (Lütken, 1874), *Pimelodus maculatus, Pimelodus pohli* Ribeiro and Lucena, 2006, *Pimelodus* sp.

**Host environments:** freshwater.

**Sites of infection:** intestine.

**Locality records:** Southeast: Minas Gerais.

**Biomes:** Cerrado.

**Specimens deposited:** vouchers CHIOC 35920, 35921.

**References:** Moreira et al. (1991); Sabas and Brasil-Sato (2014).

**Remarks:** Moreira et al. (1991) state that the type material was deposited in CHIOC, but they do not provide the codes. Additionally, Moravec (1998) considers the species to be very similar to *Spirocamallanus solani* (Pinto, Fabio, Noronha and Rolas, 1975.

*Spirocamallanus freitasi* has 17–19 spiral ridges on the buccal capsule of both sexes. Males have 9 pairs of caudal papillae (3 precloacal pairs, 1 ad-cloacal pair and 5 postcloacal pairs) and 2 unequal and dissimilar spicules. The large spicule is bifid with unequal parts: the larger part is ‘undigitated’ while the smaller part is bifid at the extremity; the smaller spicule is sharply ended (Moreira et al., 1991). *Spirocamallanus solani* has 12 spiral ridges in males and 17 spiral ridges in females; males have 9 pairs of caudal papillae (2 precloacal, 2 ad-cloacal and 5 postcloacal pairs); 2 unequal spicules with narrow alae and a simple morphology (Pinto et al., 1975). Therefore, we consider *Sp. freitasi* and *Sp. solani* to have unique morphological characteristics and validate the species as distinct taxa.

***Spirocamallanus halitrophus* Fusco and Overstreet, 1978**

**Hosts:** *Citharichthys macrops* Dresel, 1885, *Mullus argentinae* Hubbs and Marini, 1933, *Paralichthys isosceles* Jordan, 1891, *Syacium papillosum* (Linnaeus, 1758), *Urophycis brasiliensis* (Kaup, 1858), *Xystreurys rasile* (Jordan, 1891).

**Host environments:** marine.

**Sites of infection:** intestine.

**Locality records:** South: Rio Grande do Sul, Santa Catarina; Southeast: Rio de Janeiro.

**Biomes:** Atlantic Forest and Pampa.

**Specimens deposited:** CHIOC 35312-35314, 38336-38337, 38724-38735.

**References:** Cárdenas and Lanfredi (2005); Cárdenas et al. (2005); Cárdenas et al. (2012); Pereira et al. (2014); Di Azevedo and Iñiguez (2018); Fonseca et al. (2022).

**Remarks:** *Spirocamallanus halitrophus* was described parasitizing *Syacium papillosum* in the Gulf of Mexico, and Cárdenas and Lanfredi (2005) reported for the first time the species in Brazil, provenient from the type host and *Citharichthys macrops*, both from the family Cyclopsettidae. The authors also presented the first SEM analysis of *Sp. halitrophus*.

***Spirocamallanus hilarii* (Vaz and Pereira, 1934)**

**Synonyms:** *Procamallanus hilarii* Vaz and Pereira, 1934; *Procamallanus* (*Spirocamallanus*) *hilarii* Vaz and Pereira, 1934; *Procamallanus cearensis* Pereira, Dias and Azevedo, 1936; *Spirocamallanus cearensis* (Pereira, Dias and Azevedo, 1936); *Spirocamallanus incarocai* Freitas and Ibañez, 1970.

**Hosts:** *Acestrorhynchus lacustris, Acestrorhynchus microlepis* (Jardine, 1841), *Astyanax bimaculatus* (=*Astyanax bimaculatus vittatus*), *Astyanax jacuhiensis* (Cope, 1824), *Cichla monoculus, Crenicichla brasiliensis* (=*Saxatilia brasiliensis*), *Geophagus brasiliensis, Hoplias lacerdae* Miranda Ribeiro, 1908, *Hoplias malabaricus, Hoplias* aff. *malabaricus, Hypostomus commersoni* Valenciennes, 1836 (=*Plecostomus commersoni* (Valenciennes, 1836)), *Moenkhausia intermedia, Oligosarcus macrolepis* (Steindachner, 1877) (=*Acestrorhamphus macrolepis* Steindachner, 1877), *Prochilodus brevis* Steindachner, 1875, *Psalidodon fasciatus* (=*Astyanax fasciatus*), *Psalidodon* aff. *fasciatus* (=*Astyanax* aff. *fasciatus*), *Psalidodon parahybae* (Eigenmann, 1908) (=*Astyanax parahybae* Eigenmann, 1908), *Psalidodon schubarti* (Britski, 1964), (=*Astyanax bimaculatus schubarti* (Britski, 1964)), *Rhamdia quelen* (Quoy and Gaimard, 1824), *Salminus hilarii, Serrapinnus heterodon, Serrapinus piaba* (Lutken, 1875), *Steindachnerina elegans* (Steindachner, 1875 (=*Curimatus elegans* (Steindachner, 1875), *Pseudocurimata elegans*)*, Trichomycterus punctulatus* Valenciennes, 1846 (=*Pygidium punctulatum* (Valenciennes, 1846)), *Triportheus signatus*, ‘lambari amarela’ and ‘lambari de cauda vermelha’ (undetermined species).

**Host environments:** freshwater.

**Sites of infection:** body cavity, eyes, gills, gonads, intestinal cecum, intestine, kidney, mesentery, stomach, pyloric cecum.

**Locality records:** Northeast: Ceará, Paraíba; South: Paraná, Rio Grande do Sul; Southeast: Minas Gerais, Rio de Janeiro, São Paulo.

**Biomes:** Atlantic Forest, Caatinga and Pampa.

**Specimens deposited:** type material in the Helminthological Collection of the Biological Institute of São Paulo, numbers 244-A e 244-B; vouchers CHIOC 29986 a-b, 31318, 31341a-d, 31347, 32360, 32361-32364, 32366-32371, 32373, 32374, 32379, 32380, 32383, 32384, 32387, 32388, 32451, 32522 a-f, 32525, 32526 a-d, 32528, 35289, 35290, 35948.

**References:** Vaz and Pereira (1934); Pereira et al. (1936); Kloss (1966); Pinto and Noronha (1976); Kohn and Fernandes (1987); Kohn et al. (1988); Kohn et al. (1989); Rodrigues et al. (1991); Welblen and Brandão (1992); Abdallah et al. (2004); Azevedo et al. (2010); Gallas et al. (2015); Corrêa et al. (2020); Duarte et al. (2022a); Falkenberg et al. (2024); De Lima et al. (2025); Silva et al. (2025).

**Unconfirmed hosts:** *Trichomycterus piurae* (Eigenmann, 1922) in Luque et al. (2011); *Brycon orbignyanus* (Valenciennes, 1850) and *Hemisorubim platyrhynchos* in Lehun et al. (2020).

**Unconfirmed specimens deposited:** deposit codes vouchers CHIOC 39117 and 39118 in Duarte et al. (2022a).

**Remarks:** *Spirocamallanus hilarii* was redescribed by Pinto and Noronha (1976) from material deposited in CHIOC. Rodrigues et al. (1991) also redescribed the species based on specimens deposited in CHIOC and Instituto Biológico Helminthological Collection (CHIB) and compared data from specimens in previous studies related to the species *Procamallanus cearensis* Pereira, Dias and Azevedo, 1936, *Spirocamallanus incarocai* Freitas and Ibañez, 1970 and *Sp. hilarii* from both original description by Vaz and Pereira (1934) and redescription made by Pinto and Noronha (1976). *Procamallanus cearensis* was previously synonymized with *Sp. hilarii* and Rodrigues et al. (1991) also synonymized *Sp. incarocai* with *Sp. hilarii*. However, there is disagreement regarding the number of caudal papillae and ridges on the buccal capsule between these specimens.

*Procamallanus cearensis* has 13–18 spiral ridges in both sexes and the males have 7 pairs of caudal papillae (4 precloacal pairs and 3 postcloacal pairs), while *Sp. incarocai* has 14 and 16 spiral ridges in males and females, respectively, and males with 9 pairs of caudal papillae (4 precloacal pairs, 1 ad-cloacal and 4 postcloacal) (Rodrigues et al., 1991). However, *Sp. hilarii* from original description by Vaz and Pereira (1934) has 16 spiral ridges in both sexes and males with 8 pairs of caudal papillae (3 precloacal, 2 ad-cloacal and 3 postcloacal); and despite the differences in number and distribution of caudal papillae, all these 3 species have 2 short and subequal spicules with similar morphometry. Thus, until these species are revised, the synonymizing should be maintained.

***Spirocamallanus inopinatus* (Travassos, Artigas and Pereira, 1928)**

**Synonyms:** *Procamallanus inopinatus* Travassos, Artigas and Pereira, 1928; *Procamallanus (Spirocamallanus) inopinatus* Travassos, Artigas and Pereira, 1928; *Procamallanus fariasi* Pereira, 1934; *Spirocamallanus fariasi* (Pereira, 1934); *Procamallanus probus* Pinto and Fernandes, 1972; *Procamallanus wrighti* Pereira, 1935.

**Hosts:** *Acestrorhynchus falcatus* (Bloch, 1974), *Acestrorhynchus falcirostris* (Cuvier, 1819), *Acestrorhynchus heterolepis* (Cope, 1878), *Acestrorhynchus lacustris, Ageneiosus inermis* (Linnaeus, 1766), *Ageneiosus* sp., *Anodus orinocensis* (Steindachner, 1887), *Anostomoides passionis* Santos and Zuanon, 2006, *Amblydoras affinis* (Kner, 1855), *Aphanotorulus emarginatus* (Valenciennes, 1840) (=*Squaliforma emarginta* Valenciennes, 1840, *Squaliforma squalina* (Jardine, 1841)), *Arapaima gigas, Astronotus ocellatus, Astyanax altiparanae, Astyanax bimaculatus, Astyanax lacustris* (=*Astyanax bimaculatus lacustris* (Lütken, 1874)), *Astyanax* sp., *Auchenipterichthys coracoideus* (Eigenmann and Allen, 1942), *Auchenipterichthys thoracatus* (Kner, 1858), *Auchenipterus ambyiacus* Fowler, 1915, *Auchenipterus brachyurus* (Cope, 1878), *Auchenipterus nuchalis* (Spix and Agassiz, 1829), *Biotodoma cupido, Brachychalcinus copei* (Steindachner, 1882), *Brycon amazonicus* (Spix and Agassiz, 1829), *Brycon cephalus* (Günther, 1869) (=*Brycon erythropterum* (Cope, 1872)), *Brycon falcatus* Müller and Troschel (=*Brycon brevicaudatus* Günther, 1864), *Brycon hilarii* (Valenciennes, 1850), *Brycon orbignyanus, Brycon* sp., *Bryconops caudomaculatus* (Günther, 1864), *Bryconops melanurus* (Bloch, 1974), *Calophysus macropterus, Catathyridium jenynsii* (Günther, 1862), *Chalceus epakros* Zanata and Toledo-Piza, 2004, *Charax pauciradiatus* (Günther, 1864), *Cheirocerus eques* Eigenmann, 1917, *Cheirodon jaguaribensis* Fowler, 1941, *Cichla kelberi* Kullander and Ferreira, 2006, *Cichla monoculus, Cichla nigromaculata* Jardine and Schomburgk, 1843, *Cichla* sp., *Cichlasoma bimaculatum* (Linnaeus, 1758), *Colossoma macropomum, Colossoma macropomum* × *Piaractus brachypomus* (hybrid fish), *Corydoras amapaensis* Nijssen, 1972, *Corydoras ephippifer* Nijssen, 1972, *Corydoras melanistius* Regan, 1912, *Corydoras spilurus* Norman, 1926, *Crenicichla haroldoi* Luengo and Britski, 1974, *Crenicichla* sp., *Ctenobrycon* sp., *Curimatella dorsalis* (Eigenmann and Eigenmann, 1889), *Curimatella* sp., *Cynodon gibbus* (Agassiz, 1829), *Eleotris pisonis* (Gmelin, 1789), *Epapterus dispilurus* Cope, 1878, *Erythrinus erythrinus* (Bloch and Schneider, 1801), *Galeocharax humeralis* (Valenciennes, 1834) (=*Cynopotamus humeralis* Valenciennes, 1834), *Geophagus altifrons* Heckel, 1840, *Hemiodus microlepis* Kner, 1858, *Hemiodus unimaculatus* (Bloch, 1974), *Heros severus* Heckel, 1840, *Hoplerythrinus unitaeniatus, Hoplias malabaricus, Hoplias* aff. *malabaricus, Hyphessobrycon takasei* Géry, 1964, *Hypomasticus copelandii* (=*Leporinus copelandii*), *Leporellus vittatus* (Valenciennes, 1850), *Leporinus fasciatus, Leporinus friderici, Leporinus jamesi, Leporinus lacustris* (Amaral Campos, 1945), *Leporinus moralesi* Fowler, 1942, *Leporinus piau, Leporinus striatus, Leporinus taeniatus* Lütken, 1875, *Leporinus* sp., *Megaleporinus elongatus* (=*Leporinus elongatus*), *Megaleporinus macrocephalus* (=*Leporinus macrocephalus*), *Megaleporinus obtusidens* (=*Leporinus obtusidens*), *Megaleporinus reinhardti* (Lütken, 1875) (=*Leporinus reinhardti* Lütken, 1875), *Mesonauta festivus* (Heckel, 1840), *Metynnis hypsauchen* (Müller and Troschel, 1844), *Metynnis lippincottianus* (Cope, 1870), *Metynnis luna* Cope, 1878, *Moenkhausia intermedia, Myloplus arnoldi* Ahl, 1936, *Myloplus asterias* (Müller and Troschel, 1844), *Myloplus rubripinnis* (Müller and Troschel, 1844), *Mylossoma duriventre* (Cuvier, 1818), *Nemadoras humeralis* (Kner, 1855), *Ossancora asterophysa* Birindelli and Sabaj Pérez, 2011, *Pimelodina flavipinnis* Steindachner, 1876, *Pimelodus blochii, Pimelodus ornatus* Kner, 1858, *Pimelodus pictus* Steindachner, 1876, *Pimelodus* sp., *Platydoras costatus* (Linnaeus, 1758), *Potamorhina altamazonica, Potamotrygon motoro, Prochilodus lineatus, Prochilodus nigricans* Spix and Agassiz, 1829, *Proloricaria prolixa* (Isbrücker and Nijssen, 1978) (=*Loricaria prolixa* Isbrücker and Nijssen, 1978), *Propimelodus caesius* Parisi, Lundberg and DoNascimiento, 2006, *Psalidodon fasciatus, Psalidodon schubarti* (=*Astyanax bimaculatus schubarti*), *Pterodoras granulosus* (Valenciennes, 1821), *Pygocentrus nattereri, Pygocentrus piraya* (Cuvier, 1819), *Rhaphiodon vulpinus* Spiz and Agassiz, 1829, *Roeboides myersii* Gill, 1870, *Roeboides* sp., *Salminus brasiliensis* (Cuvier, 1816), *Satanoperca jurupari, Schizodon borelli, Schizodon fasciatus, Schizodon knerii* (Steindachner, 1875), *Schizodon nasutus, Semaprochilodus insignis* (Jardine, 1841), *Serrasalmus altispinis* Merckx, Jégu and Santos, 2000, *Serrasalmus brandtii* Lütken, 1875, *Serrasalmus eigenmanni* Norman, 1929, *Serrasalmus maculatus* Kner, 1858, *Serrasalmus marginatus, Serrasalmus rhombeus* (Linnaeus, 1766), *Serrasalmus spilopleura, Serrapinnus heterodon, Sorubim lima, Steindachnerina bimaculata* (Steindachner, 1876), *Sternarchella schotti* (Steindachner, 1868), *Tetragonopterus argenteus* Cuvier, 1816, *Tetragonopterus chalceus, Thoracocharax stellatus* (Kner, 1858), *Trachelyopterus galeatus, Trachydoras paraguayensis, Triportheus angulatus, Triportheus auritus* (Valenciennes, 1850), *Triportheus curtus* (Garman, 1890), *Triportheus elongatus* (Günther, 1864), *Triportheus rotundatus* (Jardine, 1841), *Triportheus signatus, Triportheus trifurcatus* (Castelnau, 1855), ‘lambari de rabo amarelo’, ‘piau’ (local names).

**Host environments:** freshwater.

**Sites of infection:** abdominal cavity, intestinal cecum, intestine, liver, mesentery, stomach, swim bladder, pyloric diverticulum.

**Locality records:** Midwest: Goiás, Mato Grosso, Mato Grosso do Sul; North: Acre, Amapá, Amazonas, Pará, Rondônia; Northeast: Bahia, Ceará, Maranhão, Paraíba, Piauí; South: Paraná; Southeast: Espírito Santo, Minas Gerais, Rio de Janeiro, São Paulo.

**Biomes:** Amazon, Atlantic Forest, Caatinga, Cerrado and Pantanal.

**Specimens deposited:** CHIOC 11463, 11467, 11468, 11471, 11473, 13055, 14634, 14644, 14652, 14762, 14729, 16496, 16506, 16509, 16645, 16525, 16575, 16800, 19743, 28460, 28474, 30601a-c, 30614a-f, 30644a-d, 31064a-r, 31091-31095, 31096a-b, 31097, 31315a-b, 31316a-c, 31324, 31325, 31326a-b, 31329, 31332, 31335, 31336a-c, 31337, 31338a-b, 31339a-b, 31343, 31348, 33331, 35556, 36950, 39133-39138, 39134, 39353, 39354; CHIBB 5008, 6809, 6813, 7871, 7954; INPA 057, 79, 80.

**References:** Travassos and Kohn (1965); Kloss (1966); Kohn et al. (1985); Kohn and Fernandes (1987); Pinto et al. (1975); Pinto and Noronha (1976); Pinto et al. (1976); Petter and Thatcher (1988); Moravec et al. (1993); Moreira et al. (1994); Machado et al. (1995); Machado et al. (1996); Andrade et al. (2001); Feltran et al. (2004); Pavanelli et al. (2004); Andrade and Malta (2006); Guidelli et al. (2006); Saraiva et al. (2006a); Saraiva et al. (2006b); Araújo et al. (2009); Moreira *et al*. (2009); Takemoto et al. (2009); Azevedo et al. (2010); Moreira et al. (2010); Kohn et al. (2011); Silva et al. (2011); Vicentin et al. (2011); Abdallah et al. (2012); Gaines et al. (2012); Mesquita et al. (2012); Vicentin et al. (2013); Gonçalves et al. (2014); Santos-Clapp and Brasil-Sato (2014); Tavares-Dias et al. (2014); Alcântara and Tavares-Dias (2015); Dias et al. (2015a); Dias et al. (2015b); Oliveira et al. (2015); Camargo et al. (2016); Hoshino et al. (2016); Oliveira et al. (2016); Pedro et al. (2016); Ribeiro et al. (2016); Moreira et al. (2017); Oliveira et al. (2017); Santos and Tavares-Dias (2017); Tavares-Dias (2017); Tavares-Dias et al. (2017); Almeida-Berto et al. (2018); Fujimoto et al. (2018); Leite et al. (2018); Morey and Malta (2018a); Morey and Malta (2018b); Oliveira et al. (2018); Pelegrini et al. (2018); Pereira et al. (2018); Baia et al. (2019); Ferreira et al. (2019); Morais et al. (2019); Negreiros *et al*. (2019); Oliveira et al. (2019); Pereira et al. (2019); Acosta et al. (2020); Ailán-Choke et al. (2020); Carvalho et al. (2020); Corrêa et al. (2020); Gião et al. (2020); Almeida et al. (2021); Borges et al. (2021); Lima et al. (2021); Negreiros et al. (2021); Virgilio et al. (2021); Alexandre and Yamada (2022); Brito-Júnior et al. (2022); Cárdenas et al. (2022); Duarte et al. (2022a); Duarte et al. (2022b); Lima et al. (2022); Santos-Clapp et al. (2022); Virgilio et al. (2022); Amaral et al. (2023); De Sousa et al. (2023); Ferreira-Cordeiro et al. (2023); Falkenberg et al. (2024); Freitas et al. (2024); Lima and Tavares-Dias (2023); Sousa et al. (2024); De Lima et al. (2025).

**Unconfirmed hosts:** *Salminus brasiliensis* (=*Salminus maxillosus* (Cuvier, 1816)) in Vicente and Pinto (1999); *Brycon orthotaenia* Günther, 1864, *Charax gibbosus* (Linnaeus, 1758), *Leporinus agassizii* Steindachner, 1876, *Leporinus octofasciatus, Myloplus schomburgkii* (Jardine, 1841) (=*Myleus schomburgkii* (Jardine, 1841)), *Prochilodus lineatus, Pygocentrus* sp., *Salminus hilarii, Serrasalmus gouldingi* Fink and Machado-Allison, 1992, *Serrasalmus manueli* (Fernández-Yépez and Ramírez, 1967) and *Triportheus paranensis* Günther, 1874 in Luque et al. (2011); *Brycon melanopterus* (Cope, 1872), *Cichla ocellaris, Cichlasoma amazonarum* Kullander, 1983, *Geophagus brasiliensis, Hemibrycon surinamensis* Géry, 1962, *Psalidodon fasciatus* (=*Astyanax fasciatus*) and *Psalidodon paranae* (Eigenmann, 1914) (=*Astyanax paranae* Eigenman, 1914) in Neves et al. (2020); *Crenicichla jaguarensis* Haseman 1911, *Hoplias* spp., *Potamotrygon amandae* and *Piaractus mesopotamicus* (Holmberg, 1887) in Lehun et al. (2020); *Brycon orthotaenia* (=*Brycon lundii* Lütken, 1875) and *Harttia duriventris* Rapp Py-Daniel and Oliveira, 2001 in Santos Reis et al. (2021).

***Spirocamallanus macaensis* (Vicente and Santos, 1972)**

**Synonyms:** *Procamallanus macaensis* Vicente and Santos, 1972; *Procamallanus* (*Spirocamallanus*) *macaensis* Vicente and Santos, 1972.

**Hosts:** *Chaetodipterus faber* (Broussonet, 1782), *Dactylopterus volitans* (Linnaeus, 1758), *Menticirrhus americanus, Micropogonias undulatus* (Linnaeus, 1766), *Nebris microps* Cuvier, 1830, *Paralonchurus brasiliensis* (Steindachner, 1875) (=*Polyclemus brasiliensis* Steindacner, 1875), *Plagioscion auratus* (Castelnau, 1855), *Stellifer brasiliensis* (Schultz, 1945), *Thyrsitops lepidopoides* (Cuvier, 1832), *Urophycis brasiliensis*, ‘cara-suja’ (local name).

**Host environments:** freshwater and marine.

**Sites of infection:** intestine.

**Locality records:** Southeast: Rio de Janeiro.

**Biomes:** Atlantic Forest.

**Specimens deposited:** type material CHIOC 30645a-e; vouchers CHIOC 30723a-d, 30794a-f, 30796a-c, 30797, 30805a-b, 30794a-b, 30795d-f, 32034a-f, 32035a-b, 33844-33846, 33849, 34107a-d, 38374–38376.

**References:** Vicente and Santos (1972); Pinto and Noronha (1976); Santos et al. (1999); Alves et al. (2004); Cordeiro and Luque (2005); Sardella et al. (2017).

**Unconfirmed hosts:** *Urophycis* sp. in Luque et al. (2011).

**Remarks:** Pinto and Noronha (1976) analysed the type material of *Sp. macaensis* deposited at CHIOC and evaluated the species’ validity. Sardella et al. (2017) redescribed *Sp. macaensis* and also reexamined the type material. Additionally, these authors examined some specimens of *Spirocamallanus pereirai* (Annereaux, 1946) reported in Brazil. They verified that part of this material corresponds to the species *Sp. macaensis*, while another part could not be identified to the species level due to poor preservation (deposit codes CHIOC 32034a-f, 32035a-b, 33844-33846, 33849, 34107a-d).

***Spirocamallanus neocaballeroi* Caballero-Deloya, 1977**

**Synonyms:** *Procamallanus* (*Spirocamallanus*) *neocaballeroi* (Caballero-Deloya, 1977).

**Hosts:** *Acestrorhynchus lacustris, Astyanax bimaculatus, Cichla monoculus, Leporinus piau, Moenkhausia costae, Poecilia vivipara* Bloch and Schneider, 1801.

**Host environments:** freshwater.

**Sites of infection:** intestine.

**Locality records:** Northeast: Paraíba; Southeast: São Paulo.

**Biomes:** Caatinga and Cerrado.

**Specimens deposited:** CHIBB 6814.

**References:** Abdallah et al. (2012); De Lima et al. (2025); Sousa et al. (2025).

**Unconfirmed hosts:** *Serrasalmus maculatus* and *Serrasalmus marginatus* in Lehun et al. (2020).

***Spirocamallanus paraensis* (Pinto and Noronha, 1976)**

**Synonyms:** *Procamallanus paraensis* Pinto and Noronha, 1976; *Procamallanus* (*Spirocamallanus*) *paraensis* Pinto and Noronha, 1976.

**Hosts:** *Hoplias malabaricus*, ‘jeju’ (local name).

**Host environments:** freshwater.

**Sites of infection:** intestine.

**Locality records:** North: Pará.

**Biomes:** Amazon.

**Specimens deposited:** holotype CHIOC 31342b, allotype 31342d, paratypes 31342a,c.

**References:** Pinto and Noronha (1976); Corrêa et al. (2024).

**Unconfirmed hosts:** *Erythrinus erythrinus* in Luque et al. (2011) and *Acestrorhynchus lacustris* in Abdallah et al. (2012).

**Remarks:** *Spirocamallanus paraensis* was described based on material deposited in CHIOC.

***Spirocamallanus pereirai* (Annereaux, 1946)**

**Synonyms:** *Procamallanus pereirai* Annereaux, 1946; *Procamallanus* (*Spirocamallanus*) *pereirai* (Annereaux, 1946).

**Hosts:** *Atlantoraja castelnaui* (Miranda Ribeiro, 1907) (=*Raja castelnaui* Miranda Ribeiro, 1907), *Micropogonias furnieri* (Desmarest, 1823), *Mullus argentinae, Paralonchurus brasiliensis*.

**Host environments:** marine.

**Sites of infection:** intestine, spiral valve.

**Locality records:** Northeast: Bahia; South: Rio Grande do Sul; Southeast: Minas Gerais, Rio de Janeiro.

**Biomes:** Atlantic Forest and Pampa.

**Specimens deposited:** vouchers CHIOC 34264, 34265a-c, 34266, 35105.

**References:** Pinto et al. (1984); Knoff et al. (2001); Ribeiro et al . (2002); Luque et al. (2003); Luque et al. (2010); Simões et al. (2025).

**Unconfirmed specimens deposited:** deposit codes CHIOC 32034a-f and 32035a-b in Pinto et al. (1984).

**Remarks:** Sardella et al. (2017) analysed materials deposited in CHIOC identified as *Sp. pereirai*, but they reallocated these specimens as *Sp. macaensis* or *Spirocamallanus* sp. The present survey for *Sp. pereirai* in Brazil refers to specimens attributed to *Sp. pereirai* that were not analysed by Sardella et al. (2017).

The study by Knoff et al. (2001) indicates that the material is only from Rio Grande do Sul (South), but, checking the CHIOC codes provided by the authors, part of the material was also collected in Minas Gerais (Southeast) (according to the data recorded in CHIOC). Additionally, the deposit codes provided by Pinto et al. (1984) are not included in the CHIOC online catalogue.

***Spirocamallanus pimelodus* (Pinto, Fabio, Noronha and Rolas, 1974)**

**Synonyms:** *Procamallanus pimelodus* Pinto, Fabio, Noronha and Rolas, 1974; *Procamallanus* (*Spirocamallanus*) *pimelodus* Pinto, Fabio, Noronha and Rolas, 1974; *Procamallanus* (*Spirocamallanus*) *intermedius* Pinto, Fabio, Noronha and Rolas, 1974; *Procamallanus intermedius* Pinto, Fabio, Noronha and Rolas, 1974; *Spirocamallanus intermedius* (Pinto, Fabio, Noronha and Rolas, 1974).

**Hosts:** *Bujurquina cordemadi* Kullander, 1986, *Heros severus, Iheringichthys labrosus* (Lütken, 1874), *Nemadoras humeralis, Pimelodella lateristriga, Pimelodus blochii, Pimelodus maculatus, Pimelodus ortmanni, Pimelodus pohli, Serrasalmus maculatus, Solea senegalensis* Kaup, 1858, *Synodontis clarias* (=*Pimelodus clarias*).

**Host environments:** freshwater.

**Sites of infection:** intestine.

**Locality records:** Midwest: Mato Grosso; North: Acre; South: Paraná; Southeast: Minas Gerais, São Paulo.

**Biomes:** Amazon, Atlantic Forest and Cerrado.

**Specimens deposited:** holotype CHIOC 30993, allotype 30999, paratypes 30989a-b, 30990, 30991, 30992a-b, 30993b-i, 30994, 30995a-b, 30996, 30997a-b, 30998a-c, 30999b-f, 31000a-c, 31001, 31002a-b, 31003a-b, 31004a-c, 31005a-c, 31006a-d, 31007, 31008a-b, 31009a-c, 31010, 31011; voucher CHIOC 31022a, 31023a, 31022 b-h, 31023 b-c, 32024, 31025a-b (type series of *Spirocamallanus intermedius*); CHIOC 35918.

**References:** Kohn and Fernandes (1987); Kohn et al. (1988); Pinto et al. (1974); Moravec et al. (1993); Moreira et al. (2005); Takemoto et al. (2009); Kohn et al. (2011); Sabas and Brasil-Sato (2014); Negreiros et al. (2018); Cavalcante et al. (2020); Virgilio et al. (2022); Negreiros et al. (2024).

**Unconfirmed hosts:** *Pimelodus* sp. in Luque et al. (2011).

**Remarks:** Pinto et al. (1974) described the species *Sp. pimelodus* and *Sp. intermedius* Pinto, Fabio, Noronha and Rolas, 1974 in the same study, both parasitizing the intestine of the same host species, *Synodontis clarias* (=*Pimelodus clarias*), and Moravec et al. (1993) synonymized them, considering that the differences highlighted between the species described by Pinto et al. (1974) are not sufficient to differentiate the 2 species.

Analysing the descriptions of both species, the main characteristic to differentiate *Sp. intermedius* was the morphology of the larger spicule, which is bifurcated at its distal end and divided into zones. In contrast, the spicule of *Sp. pimelodus* has a pointed tip, and this morphological difference should be sufficient to separate the 2 species.

However, we analysed the type series of *Sp. intermedius* (codes CHIOC 31022a-h, 31023a-c). The larger spicule of this species has a pointed end, and no bifurcation was observed. Additionally, we also analysed specimens of *Sp. pimelodus* (codes CHIOC 30993b-i, 30999 b-f). The morphology of males and females is similar to *Sp. intermedius*. Thus, we agree that both species should be synonymized as proposed by Moravec et al. (1993).

Moravec et al. (1993) discuss the validity of *Sp. intermedius* because they found camallanid specimens that they identified as *Sp. pimelodus*. The authors state that their material most closely resembles *Sp. intermedius*, and after analysing the work of Pinto et al. (1974), proposed the synonym. However, evaluating the description of these specimens from Moravec et al. (1993), there are many differences between this material and the description of *Sp. pimelodus* made by Pinto et al. (1974).

The specimens identified by Moravec et al. (1993) as *Sp. pimelodus* have 11 pairs of caudal papillae (2 pairs ad-cloacal, absent in the original description of *Sp. pimelodus*), 2 unequal and dissimilar spicules, with the larger one bifurcated at its distal end and larger in size than the *Sp. pimelodus* described by Pinto et al. (1974), and the posterior end of the male has 2 mucrons (absent in the original description of the species). Thus, the identification by Moravec et al. (1993) is mistaken, and the specimens may represent another taxon.

***Spirocamallanus pintoi* Kohn and Fernandes, 1988**

**Hosts:** *Biotodoma cupido, Corydoras multiradiatus* (Orcés V., 1960) (= *Brochis multiradiatus* (Orcés V., 1960)), *Corydoras paleatus* (Jenyns, 1842), *Corydoras trilineatus* Cope, 1872.

**Host environments:** freshwater.

**Site of infections:** intestine.

**Locality records:** North: Acre; South: Paraná.

**Biomes:** Amazon and Atlantic Forest.

**Specimens deposited:** holotype CHIOC 32432a, allotype 32431a, paratypes 32431b, 32432b-c.

**References:** Kohn and Fernandes (1988a); Kohn et al. (1988); Ito et al. (2005); Virgilio et al. (2022).

**Unconfirmed hosts:** *Corydoras aeneus* (Gill, 1858) in Luque et al. (2011).

***Spirocamallanus rebecae* Andrade-Salas, Pineda-Lopez and Garcia-Magana, 1994**

**Hosts:** *Proloricaria prolixa* (=*Loricaria prolixa*), *Triportheus angulatus*.

**Host environments:** freshwater.

**Sites of infection:** intestine.

**Locality records:** Southeast: São Paulo.

**Biomes:** Cerrado.

**Specimens deposited:** CHIBB 6832, 7952.

**References:** Abdallah et al. (2012); Pelegrini et al. (2018).

***Spirocamallanus solani* (Pinto, Fabio, Noronha and Rolas, 1975)**

**Synonyms:** *Procamallanus* (*Spirocamallanus*) *solani* Pinto, Fabio, Noronha and Rolas, 1975; *Procamallanus solani* Pinto, Fabio, Noronha and Rolas, 1975.

**Hosts:** ‘bagre’ (undetermined species).

**Host environments:** freshwater.

**Sites of infection:** intestine.

**Locality records:** North: Pará.

**Biomes:** Amazon.

**Specimens deposited:** holotype CHIOC 31071a, allotype CHIOC 31062b, paratypes CHIOC 31062a-c, 31071b.

**References:** Pinto et al. (1975).

**Remarks:** *Spirocamallaus solani* was described based on material deposited in CHIOC.

***Spirocamallanus* sp.**

**Synonyms:** *Procamallanus* (*Spirocamallanus*) sp.

**Hosts:** *Anodus elongatus* Agassiz, 1829, *Arapaima gigas, Astyanax bimaculatus, Atlantoraja castelnaui* (=*Raja castelnaui*), *Auchenipterus nuchalis, Auchenipterus osteomystax* (Miranda Ribeiro, 1918)*, Brachychalcinus copei, Biotodoma cupido, Bivibranchia fowleri* (Steindachner, 1908), *Bivibranchia notata* Vari and Goulding, 1985, *Bivibranchia velox* (Eigenmann and Myers, 1927), *Brycon pesu* Müller and Troschel, 1845, *Brycon* sp., *Chaetodipterus faber, Charax pauciradiatus, Cheirodon jaguaribensis, Colossoma macropomum, Conorynchus conirostris, Corydoras armatus* (Günther, 1868), *Crenicichla brasiliensis* (=*Saxatilia brasiliensis*), *Cynopotamus kincaidi* (Schultz, 1950)*, Hemiodus microlepis, Hemiodus unimaculatus, Hydromedusa tectifera* Cope, 1870, *Loricariichthys derbyi* Fowler, 1915, *Lutjanus synagris* (Linnaeus, 1758), *Micropogonias undulatus, Moenkhausia intermedia, Paralonchurus brasiliensis, Plagioscion auratus, Pimelodus pohli, Porichthys porosissimus* (Cuvier, 1829), *Prochilodus lineatus (=Prochilodus scrofa), Psalidodon fasciatus* (=*Astyanax fasciatus*), *Pseudoplatystoma corruscans* (Spiz and Agassiz, 1829), *Raphiodon vulpinus, Roeboides affinis* (Günther, 1868), *Salminus hilarii; Satanoperca jurupari, Serrapinnus heterodon, Serrapinus piaba, Symphurus tesselatus* (Quoy and Gaimard, 1824), *Tetragonopterus chalceus, Triportheus nematurus* (=*Chalcinus nematurus*), *Triportheus guentheri, Urophycis* sp., ‘cará-cachimbo’, ‘jaruatama’, ‘matrinchã’, ‘peixe-cachorro’ (popular names).

**Host environments:** freshwater and marine.

**Sites of infection:** intestine, mesentery.

**Locality records:** Midwest: Mato Grosso; North: Acre, Amazonas, Pará, Roraima; Northeast: Ceará, Maranhão; South: Paraná, Rio Grande do Sul; Southeast: Minas Gerais, Rio de Janeiro, São Paulo.

**Biomes:** Amazon, Atlantic Forest, Caatinga and Cerrado.

**Specimens deposited:** CHIOC 30794c-b, 30796d-f, 30797, 30968a-d, 31077a-b, 31344, 31345, 31346a-b, 33848, 34624, 34625a-c, 34266, 35718, 35919, 43109, 43110.

**References:** Pinto and Noronha (1976); Pinto et al. (1976); Santos et al. (1979); Thatcher (1981); Kohn and Fernandes (1987); Kohn et al. (1988); Machado et al. (1995); Machado et al. (1996); Fischer et al. (2003); Pavanelli et al. (2004); Brasil-Sato and Santos (2005); Costa and Camargo (2009); Takemoto et al. (2009); Tavernari et al. (2009); Kohn et al. (2011); Melo et al. (2011); Novelli et al. (2014); Sabas and Brasil-Sato (2014); Albuquerque et al. (2016); Sardella et al. (2017); Cárdenas et al. (2022); Gama et al. (2022); Virgilio et al. (2022); Cárdenas et al. (2024); Falkenberg et al. (2024); Negreiros et al. (2024); Santos et al. (2024).

**Unconfirmed hosts:** *Astyanax bimaculatus, Hemiodus* sp., *Leporinus friderici, Megaloporinus obtusidens* (=*Leporinus obtusidens*), *Pachyurus squamipennis* Agassiz, 1831, *Paracheirodon axelrodi, Pimelodus maculatus, Rhinodoras dorbignyi*, ‘chimboré’ and ‘ferreirinha’ (popular names) in Luque et al. (2011); *Geophagus brasiliensis* in Lehun et al. (2020).

**Remarks:** All the studies mentioned identified camallanids only at the genus level. However, many authors use the presence of spiral ridges as a criterion for classifying specimens in the genus *Spirocamallanus*, while ignoring other structures, such as the tooth-like structures in *Denticamallanus*, whose new diagnosis is presented in this study. Therefore, these specimens identified as ‘*Spirocamallanus* sp.’ may belong to other genera, especially *Denticamallanus*, following the new taxonomic key for the family Camallanidae proposed here.

Additionally, among the hosts, *Hydromedusa tectifera* is the only chelonian recorded as a host of *Spirocamallanus*.

**Subfamily Camallaninae Yeh, 1960**

**Genus *Camallanus* Railliet and Henry, 1915**

***Camallanus acaudatus* Ferraz and Thatcher, 1990**

**Synonyms:** *Camallanus* (*Camallanus*) *acaudatus* Ferraz and Thatcher, 1990.

**Hosts:** *Osteoglossum bicirrhosum* (Cuvier, 1829).

**Host environments:** freshwater.

**Sites of infection:** large intestine, pyloric caeca, small intestine.

**Locality records:** North: Amapá, Amazonas.

**Biomes:** Amazon.

**Specimens deposited:** holotype and allotype in INPA (number not provided); paratypes CHIOC 32557.

**References:** Ferraz and Thatcher (1990); Rodrigues et al. (2014).

**Remarks:** Ferraz and Thatcher (1990) report that the type material was deposited in INPA and CHIOC, but they do not provide the codes. The CHIOC code of paratypes was obtained by consulting the CHIOC online catalogue.

***Camallanus cotti* Fujita, 1927**

**Synonyms:** *Camallanus zacconis* Li, 1941; *Camallanus fotedari* Raina and Dhar, 1972; *Camallanus maculatus* Martins, Garcia, Piazza and Ghiraldelli, 2007.

**Hosts:** *Betta splendens* Regan, 1910, *Poecilia reticulata* Peters, 1859, *Xiphophorus maculatus* (Günther, 1866).

**Host environments:** freshwater.

**Sites of infection:** intestine, rectum.

**Locality records:** Southeast: Rio de Janeiro, São Paulo.

**Biomes:** Atlantic Forest and Cerrado.

**Specimens deposited:** vouchers CHIOC 33900, 35283, 35442-35445, 36631a-b, 36632a-h, 36637a-b, 36638.

**References:** Alves et al. (2000); Menezes et al. (2006); Martins et al. (2007).

**Unconfirmed hosts:** *Apistogramma cacatuoides* Hoedeman, 1851, *Poecilia latipinna* (Lesueur, 1821), *Pterophylum scalare* (Schultze, 1823) and *Xiphophorus helleri* Heckel, 1848 in Eiras et al. (2016).

**Remarks:** Santos and Moravec (2009) reexamined the type material of *C. maculatus* Martins, Garcia, Piazza and Ghiraldelli, 2007 and synonymized this species with *C. cotti*. This synonymy had already been suggested by Moravec and Justine (2006); however, the description article of *C. maculatus* had not yet been published.

***Camallanus tridentatus* (Drasche, 1884)**

**Synonyms:** *Cucullanus tridentatus* Drasche, 1884; *Camallanus* (*Camallanus*) *tridentatus* (Drasche, 1884).

**Hosts:** *Arapaima gigas*.

**Host environments:** freshwater.

**Sites of infection:** intestine, pyloric caecum, stomach.

**Locality records:** North: Amazonas, Pará.

**Biomes:** Amazon.

**Specimens deposited:** vouchers in INPA (number not provided), CHIOC 32556, 35611.

**References:** Ferraz and Thatcher (1990); Araújo et al. (2009); Santos and Moravec (2009).

**Remarks:** Ferraz and Thatcher (1990) report that the voucher specimens were deposited in INPA and CHIOC, but they do not provide the codes. The CHIOC code was obtained by consulting the CHIOC online catalogue. Ferraz and Thatcher (1990) described the male of the species, which had been described by Drasche (1884) based only on the female. Additionally, Santos and Moravec (2009) redescribed *C. tridentatus* and conducted the first SEM analysis of the species.

***Camallanus* sp.**

**Hosts:** *Acanthochelys spixii* (Duméril and Bibron, 1835), *Astronotus ocellatus, Bothrops atrox* (Linnaeus, 1758), *Charax gibbosus, Corydoras ephippifer, Corydoras melanistius, Hydromedusa tectifera, Hyphessobrycon takasei, Hyphessobrycon amapaensis* Zarske and Géry, 1998, *Phrynops hilarii* (Duméril and Bibron, 1835), *Phrynops geoffroanus* (Schweigger, 1812), *Pseudoplatystoma fasciatum* (Linnaeus, 1766), *Trachemys dorbigni* (Duméril and Bibron, 1835).

**Host environments:** freshwater and terrestrial.

**Sites of infection:** coelomic cavity, large intestine, mesentery, small intestine, stomach.

**Locality records:** Midwest: Mato Grosso do Sul; North: Amapá, Pará, Rondônia; Northeast: Paraíba; South: Rio Grande do Sul; Southeast: Minas Gerais.

**Biomes:** Amazon, Atlantic Forest and Pampa.

**Specimens deposited:** CHIOC 38320.

**References:** Travassos and Freitas (1942); Bernadon et al. (2013); Mascarenhas et al. (2013); Bernadon et al. (2014); Vieira et al. (2016); Ferreira et al. (2019); Pereira et al. (2019); Mascarenhas et al. (2022); Conga et al. (2024); Freitas et al. (2024); Izidro de Brito and Figueiredo Lacerda (2025).

**Remarks:** Conga et al. (2024) presented the first official record of Camallanidae parasitizing snakes (*Bothrops atrox*) in Brazil.

**Genus *Oncophora* Diesing, 1851**

***Oncophora melanocephala* (Rudolphi, 1819)**

**Synonyms:** *Cucullanus melanocephalus* Rudolphi, 1819; *Trichocephalus gibbosus* Rudolphi, 1819; *Oncophora neglecta* Diesing, 1851; *Oncophora albacarensis* Baudin-Laurencin, 1972.

**Hosts:** *Auxis thazard* (Lacepède, 1800), *Priacanthus arenatus* Cuvier, 1829, *Sarda sarda, Thunnus albacares* (Bonnaterre, 1788), *Thunnus atlanticus* (Lesson, 1831), *Thunnus thynnus* (Linnaeus, 1758).

**Host environments:** marine.

**Sites of infection:** intestine, pyloric organ, small intestine, stomach.

**Locality records:** Southeast: Rio de Janeiro.

**Biomes:** Atlantic Forest.

**Specimens deposited:** voucher in the Institute of Parasitology, Academy of Sciences of the Czech Republic (ASCR), Ceske Budejovice n-712, CHIOC 32248a-b, 32249a-b, 32250, 32251a-b, 32331, 33967-33971, 35377.

**References:** Moravec et al. (1999); Pinto et al. (1988); Tavares et al. (2001); Alves and Luque (2006).

**Remarks:** Baudin-Laurencin (1971) redescribed *Cucullanus melanocephalus* Rudolphi, 1819, a parasite of *Thunnus albacares* (Bonnaterre, 1788) (=*Neothunnus albacares*), and reallocated the species to the genus *Oncophora*, named as *Oncophora melanocephala*. Additionally, Pinto et al. (1988) and Moravec et al. (1999) redescribed *O. melanocephala*, highlighting that this is the only valid species of the genus.

Due to the question of the synonymy between the genera *Oncophora* and *Paracamallanus* Yorke and Maplestone, 1926 in the literature, we evaluated specimens of *O. melanocephala* deposited in CHIOC (codes CHIOC 32348a, 32248b, 32249a, 32349b and 33967) and specimens of *Paracamallanus* (see below). We observed that *Oncophora* and *Paracamallanus* are distinct genera. The only similarity between the 2 genera is the division of the buccal capsule into 3 chambers. However, *Oncophora* has the first chamber twice as large as the next 2. Additionally, the first 2 chambers are very sclerotized, giving them a dark coloration, almost black, while the third is very hyaline, and the tridents of *Oncophora* are more elongated.

**Genus *Paracamallanus* Yorke and Maplestone, 1926**

***Paracamallanus amazonensis* Ferraz and Thatcher, 1992**

**Hosts:** *Hypophthalmus edentatus* Spix and Agassiz, 1829, *Plagioscion squamosissimus* (Heckel, 1840), *Pterodoras granulosus*.

**Host environments:** freshwater.

**Sites of infection:** intestine.

**Locality records:** North: Amazonas; South: Paraná.

**Biomes:** Amazon and Atlantic Forest.

**Specimens deposited:** type material in INPA (number not provided); vouchers CHIOC 32945, 32948, 32956, 32957.

**References:** Ferraz and Thatcher (1992); Moravec et al. (1993); Kohn et al. (2011).

**Unconfirmed hosts:** *Hypophthalmus oremaculatus* Nani and Fuster de Plaza, 1947, and *Piaractus mesopotamicus* in Lehun et al. (2020).

**Remarks:** Ferraz and Thatcher (1990) report that the type material was deposited in INPA, but they do not provide the codes. Additionally, we evaluated specimens of *Pa. amazonensis* deposited at CHIOC (codes CHIOC 32945, 32948, 32956 and 32957) and corroborated that the genus *Paracamallanus* is a distinct taxon when compared to *Oncophora. Paracamallanus* also has a buccal capsule divided into 3 chambers; however, the first 2 chambers are of similar size. In addition, the buccal capsule of *Paracamallanus* is brown-orange.

**Genus *Serpinema* Yeh, 1960**

***Serpinema amazonicum* (Ribeiro, 1941)**

**Synonyms:** *Serpinema amazonicus* (Ribeiro, 1941); *Camallanus amazonicus* Ribeiro, 1941.

**Hosts:** *Mesoclemmys tuberculata* (Luederwaldt, 1926), *Phrynops geoffroanus, Podocnemis expansa* (Schweigger, 1812).

**Host environments:** freshwater/terrestrial.

**Sites of infection:** intestine, large intestine, lungs.

**Locality records:** North: Pará; Northeast: Sergipe.

**Biomes:** Amazon and Caatinga.

**Specimens deposited:** type material CHIOC 13722.

**References:** Ribeiro (1941); Santana et al. (2023).

**Remarks:** Ribeiro (1941) states that the type material was deposited in CHIOC but does not provide the code. The code was obtained by consulting the CHIOC online catalogue. The author suggests that *Se. amazonicu*m (=*C. amazonicus*) is probably the same species identified as *Serpinema microcephalus* (Dujardin, 1845) (=*Cucullanus microcephalus*) by Dujardin (1845) and *Serpinema trispinosum* (Leidy, 1852) (=*C. trispinosus*) by Railliet and Henry.

However, Silva et al. (2023) provide a compilation with morphometric and morphological data of the valid species of *Serpinema*, and *Se. amazonicum* has a greater number of buccal capsule ridges, fewer pairs of caudal papillae, smaller spicules and longer specimens when compared to *Se. microcephalus*. Also, *Se. amazonicum* has more ridges on the buccal capsule, fewer pairs of caudal papillae, smaller spicules and shorter specimens when compared to *Se. trispinosum*. Thus, we accepted the validity of *Se. amazonicum*.

Additionally, the generic name *Serpinema* is a neutral word; however, the specific epithet ‘*amazonicus*’ is not. Therefore, following the International Code of Zoological Nomenclature (2012), we proposed the change of the epithet to ‘*amazonicum*’ to match the gender of the genus.

***Serpinema emydidium* (Mascarenhas and Müller, 2017)**

**Synonyms:** *Camallanus emydidius* Mascarenhas and Müller, 2017.

**Hosts:** *Hydromedusa tectifera, Trachemys dorbigni*.

**Host environments:** freshwater/terrestrial.

**Sites of infection:** small intestine.

**Locality records:** South: Rio Grande do Sul.

**Biomes:** Pampa.

**Specimens deposited:** holotype and allotype CHLAPASIL-UFPel 285-286, paratypes CHLAPASIL-UFPel 523-527 and CHIOC 38507-38512; vouchers CHLAPASIL 306, 313, 314, 316, 779.

**References:** Mascarenhas and Müller (2017); Chaviel et al. (2020); Mascarenhas et al. (2022).

**Remarks:** Mascarenhas and Müller (2017) described *Se. emydidium* (=*C. emydidius*) parasitizing the freshwater turtle *Hydromedusa tectifera*; however, they allocated the species into the genus *Camallanus*. The line drawings and photomicrographs in their study illustrate the presence of a gap between the longitudinal ridges of the buccal capsule in the specimens. Usually, the length of the incomplete median ridges is inconsistent and more elongated in some specimens. However, we clearly observed a gap between the ridges (the main characteristic of *Serpinema*). Therefore, we reallocated the species to the genus *Serpinema*.

Following the International Code of Zoological Nomenclature (2012) we change the specific epithet to ‘*emydidium*’ to match the gender of the genus *Serpinema*, which is a neutral word.

***Serpinema magathi* (Sprehn, 1932)**

**Synonyms:** *Camallanus magathi* Sprehn, 1932, *Camallanus parvus* Caballero, 1939.

**Hosts:** *Kinosternon scorpioides* (Linnaeus, 1766).

**Host environments:** freshwater/terrestrial.

**Sites of infection:** large intestine, small intestine, stomach.

**Locality records:** North: Pará; Northeast: Maranhão, Pernambuco.

**Biomes:** Amazon and Caatinga.

**Specimens deposited:** vouchers in the Anatomy Laboratory of the State University of Maranhão, access number 001/2014, MPEG number 11-16, CHIOC 28262a-e, 28263a-c, 28264.

**References:** Alho (1965); Freitas and Dobbin (1971); Viana et al. (2016).

**Remarks:** *Serpinema magathi* (=*C. magathi*) was insufficiently described into the genus *Camallanus* by Sprehn (1932), parasitizing *Kinosternon scorpioides* (=*Kinosternon scorpioides integrum*) from Bolivia. However, later, Freitas and Dobbin (1971) provided a complementary description and illustrations for the species. Additionally, Silva et al. (2023) reexamined the voucher specimens deposited in CHIOC of the study by Freitas and Dobbin (1971) (CHIOC 28262a-c, 28263 a-b, 28264) and reinforce the validity of the species.

***Serpinema microcephalus* (Dujardin, 1845)**

**Synonyms:** *Camallanus microcephalus* (Dujardin, 1845); *Cucullanus microcephalus* Dujardin, 1845; *Serpinema microcephalum* (Dujardin, 1845).

**Hosts:** *Podocnemis expansa, Podocnemis unifili*s Troschel, 1848 (=*Podocnemis* ‘Tracaca’).

**Host environments:** freshwater/terrestrial.

**Sites of infection:** small intestine.

**Locality records:** Brazil (locality not specified).

**Biomes:** undetermined.

**References:** Diesing (1851).

**Unconfirmed hosts:** *Peltocephalus dumerilianus* (Schweigger, 1812) in Mascarenhas and Müller (2021) and Izidro de Brito and Figueiredo Lacerda (2025).

**Remarks:** Diesing (1851) reported that *Se. microcephalus* (=*Cucullanus microcephalus*) was recorded in *Podocnemis expansa* and ‘*Podocnemis* Tracaca’ (as the author wrote). The term ‘Tracaca’ corresponds to ‘Tracajá’, the common name for the freshwater turtle *Podocnemis unifilis*. Therefore, we infer that *Podocnemis unifilis* is also a host species for *Se. microcephalus*.

We believe that at some point of studies conducted with these nematodes there was a confusion in reporting the hosts of *Se. microcephalus* in Brazil. Both checklists elaborated by Mascarenhas and Müller (2021) and Izidro de Brito and Figueiredo Lacerda (2025) report *Peltocephalus dumerilianus* as host of this nematode, citing Diesing (1851) as the original reference. However, as we have indicated, Diesing (1851) does not mention this species of freshwater turtle as a host for *Se. microcephalus*.

***Serpinema monospiculatum* Freitas and Dobbin Jr., 1962**

**Synonyms:** *Serpinema monospiculatus* Freitas and Dobbin Jr., 1962.

**Hosts:** *Mesoclemmys nasuta* (Schewwigger, 1812) (=*Batrachemys nasuta* (Schweigger, 1812)), *Mesoclemmys tuberculata* (=*Batrachemys tuberculata* (Luederwaldt, 1926), *Phrynops geoffroanus*.

**Host environments:** freshwater/terrestrial.

**Sites of infection:** body cavity, lung, large intestine, small intestine, stomach.

**Locality records:** Northeast: Ceará, Pernambuco, Sergipe.

**Biomes:** Caatinga.

**Specimens deposited:** holotype CHIOC 28265a, allotype 28265b, paratypes 28264c-I; vouchers CHIOC 28266, 28267a-c, 28268a-b, 28269a-b, 28270, 28271a-c, 28272.

**References:** Freitas and Dobbin (1971); Pereira et al. (2018); Fonseca et al. (2021); Santana et al. (2023).

**Remarks:** Silva et al. (2023) reexamined specimens of *Se. monospiculatum* deposited in CHIOC and corroborated the validity of the species. Also, the generic name *Serpinema* is a neutral word; however, the specific epithet ‘*monospiculatus*’ is not. Therefore, following the International Code of Zoological Nomenclature (2012), we proposed changing the epithet to ‘*monospiculatum*’ to match the gender of the genus.

***Serpinema pelliculatum* Silva, Jesus and Melo, 2023**

**Synonyms:** *Serpinema pelliculatus* Silva, Jesus and Melo, 2023.

**Hosts:** *Kinosternon scorpioides*.

**Host environments:** freshwater/terrestrial.

**Sites of infection:** intestine, stomach.

**Locality records:** North: Pará.

**Biomes:** Amazon.

**Specimens deposited:** holotype MPEG.PLA 000399, allotype MPEG.PLA 000400, paratypes MPEG.PLA 000401, MPEG.PLA 000402.

**References:** Silva et al. (2023).

**Unconfirmed locality records:** Roraima (North) in Izidro de Brito and Figueiredo Lacerda (2025).

**Remarks:** The generic name *Serpinema* is neutral; however, the specific epithet ‘*pelliculatus*’ of this species is not. Therefore, following the International Code of Zoological Nomenclature (2012), we proposed the change of the epithet to ‘*pelliculatum*’ to match the gender of the genus.

***Serpinema trispinosum* (Leidy, 1852)**

**Synonyms:** *Camallanus magnorugosus* Caballero, 1939, *Camallanus pipientis* Walton, 1935, *Cucullanus trispinosus* Leidy, 1852, *Serpinema magnorugosus* (Caballero, 1939), *Serpinema magnorugosum* (Caballero, 1939), *Serpinema trispinosus* (Leidy, 1852).

**Hosts:** *Astyanax bimaculatus, Astronotus ocellatus, Cheirodon jaguaribensis, Cichla monoculus, Cichlasoma orientale* Kullander, 1983, *Crenicichla brasiliensis, Leporinus taeniatus, Moenkhausia intermedia, Poecilia vivipara, Psalidodon fasciatus, Serrapinnus heterodon, Serrapinnus piaba, Triportheus signatus*.

**Host environments:** freshwater.

**Sites of infection:** intestine, mesentery.

**Locality records:** Northeast: Ceará.

**Biomes:** Caatinga.

**References:** Falkenberg et al. (2024).

**Remarks:** Falkenberg et al. (2024) presented the first record of *Se. trispinosum* in Brazil, but the authors found only larval stages.

**Unconfirmed species report**

***Spirocamallanus paraguayensis* Petter, 1990**

**Synonyms:** *Procamallanus* (*Spirocamallanus*) *paraguayensis* (Petter, 1990).

**Hosts:** *Brycon orbignyanus.*

**Host environments:** freshwater.

**Sites of infection:** intestine.

**Locality records:** South: Paraná.

**Biomes:** Atlantic Forest.

**References:** Lehun et al. (2020).

**Remarks:** Lehun et al. (2020) presented an updated checklist of fish parasites from the Upper Paraná River floodplain, reporting the occurrence of *Sp. paraguayensis* in *Brycon orbignyanus*. However, upon reviewing the reference list of that study, we were unable to identify any publication that formally documents this record. Furthermore, in our entire survey, we did not identify any study reporting this species in Brazil. Therefore, we consider invalid the record of *Sp. paraguayensis* in Brazil indicated by Lehun et al. (2020), and we conclude that the species has not yet been reported in the country.

***Paracamallanus* sp.**

**Hosts:** *Hypophtalmus edentatus*.

**Host environments:** freshwater.

**Sites of infection:** not informed.

**Locality records:** Amazonia (Brazil).

**Biomes:** Amazon.

**References:** Thatcher (1991).

**Remarks:** Thatcher (1991) published the book ‘Amazon Fish Parasites’, in which he listed fish parasite species recorded in the Amazon region. However, no references were provided. Notably, in our entire survey, this is the only report of the occurrence of *Paracamallanus* sp. Therefore, we consider invalid the record of *Paracamallanus* sp. in Brazil indicated by Thatcher (1991).

**Species *inquirenda***

***Spirocamallanus barroslimai*** **(Pereira, 1935)**

**Synonyms:** *Procamallanus barroslimai* Pereira, 1935; *Procamallanus* (*Spirocamallanus*) *barroslimai* Pereira, 1935.

**Hosts:** Clupeidae gen sp. (popular name: ‘sardinha’).

**Host environments:** freshwater.

**Sites of infection:** intestine.

**Locality records:** Northeast: Rio Grande do Norte.

**Biomes:** Atlantic Forest.

**References:** Pereira (1935); Pinto and Noronha (1976).

**Unconfirmed hosts:** *Triportheus* sp. in Thatcher (2006).

**Unconfirmed locality records:** Amazonas (North) in Luque et al. (2011).

**Remarks:** Pereira (1935) described *Sp. barroslimai* and allocated the species into the genus *Procamallanus* as parasitizing the fish ‘sardinha’ (local name), based only on the male of the species. Later, Pinto and Noronha (1976) suggested that *Sp. barroslimai* should be synonymized with *Sp. inopinatus* due to the similarities between the species. However, they could not propose the synonymy because they analysed only a male specimen and were unable to access the type material. The female of the species remains unknown, and the description and illustrations of the male are insufficient to allow proper identification of the species. Thus, we consider *Sp. barroslimai* a species *inquirenda*.

## Discussion

The predominant presence of fish as hosts of camallanids is related to the life cycle of these nematodes. Definitive hosts, including fish and reptiles, shed these helminth larvae in their feces. Copepods then ingest this larva, which develops to the next stage, acting as an intermediate host. The cycle completes when the definitive host consumes the infected copepods, or it may involve paratenic hosts, such as small fish or amphibians. When the definitive host ingests an infected paratenic host, the camallanid larvae resume their cycle, reaching the adult stage (Stromberg and Crites, 1974a; Bartlett and Anderson, 1985; Moravec, 1994, 1998; González and Hamman, 2007; Wiles and Bolek, 2015).

Furthermore, it is worth noting that studies on the helminth fauna of fish in Brazil are more common than similar studies on other vertebrate groups, such as turtles and snakes (Borges et al., 2018; Lehun et al., 2020; Mascarenhas and Müller, 2021). Although studies of anuran parasites are also very common in Brazil (Campião et al., 2014), there are still no records of camallanids in amphibians in the country. This fact is particularly notable considering that camallanid infections in amphibians, although relatively uncommon, have been reported in other regions of the world (Svitin et al., 2019; Bursey and Goldberg, 2020), including South America (González and Hamman, 2007; González et al., 2021).

To organize our checklist, we first created a new key to identify the subfamilies and genera of the family Camallanidae. Based on the buccal capsule morphology, we recognized only 2 subfamilies (Camallaninae and Procamallaninae). Stromberg and Crites (1974b) proposed the subfamily Paracamallaninae Stromberg and Crites, 1974, based on the buccal capsule morphology, composed of 2 chambers, to allocate the genus *Paracamallanus*. However, we do not consider this subfamily valid because the genera within Paracamallaninae share a key morphological trait, a buccal capsule formed by 2 valves, which is characteristic of the subfamily Camallaninae.

Moravec and Scholz (1991), Moravec and Thatcher (1997), and, more recently, Gupta (2024) proposed keys for identifying subgenera within the genus *Procamallanus*. However, we disagree with this classification and emphasize that there is no supporting evidence for the maintenance of subgenera within Camallanidae, as noted by Ailán-Choke and Pereira (2021). Therefore, the new taxonomic key proposed here recognizes 13 valid genera within Camallanidae.

Vences et al. (2013) emphasized that subgeneric classification should be applied when molecular data for a group are limited or when phenotypic diagnoses are poorly defined. Although the family Camallanidae meets the first criterion, the taxa traditionally considered as subgenera exhibit well-defined diagnostic features, particularly in the morphology and distribution pattern of internal ornamentations within the buccal capsule. Additionally, phylogenetic evidence supports the recognition of generic status for some of these taxa (Ailán-Choke and Pereira, 2021), including *Camallanus, Procamallanus, Serpinema* and *Spirocamallanus*; and even *Denticamallanus* (see below).

Following the proposed classification, we reallocated 8 species. We transferred 2 species of *Procamallanus* and 5 species of *Spirocamallanus* to the genus *Denticamallanus*. These included *D. annipetterae, D. spiculstriatus* (both previously in *Procamallanus*), *D. chimuensis, D. iheringi, D. krameri, D. rarus* and *D. saofranciscensis* (all previously in *Spirocamallanus*). Additionally, we reallocated 1 *Camallanus* specie*s* to *Serpinema*, namely *Se. emydidium*.

To date, only 2 species of *Denticamallanus* were known: *D. dentatus* and *D. ana* Ramallo, 2011 (Moravec and Thatcher, 1997; Ramallo, 2011). However, with this checklist, the genus now includes 9 valid species. Moravec and Thatcher (1997) initially proposed *Denticamallanus* as a subgenus of *Procamallanus*. The main characteristic was the presence of a rounded buccal capsule with conical internal projections similar to teeth in males, and saw-shaped spiral ridges in females, with both structures in the posterior region of the buccal capsule.

We propose here a new diagnosis for the genus *Denticamallanus*, based on the presence of tooth-like structures in the buccal capsule of at least 1 sex. These structures may be present in both sexes in some species, and spiral ridges may be present or absent. Thus, we consider the presence of tooth-like structures at the basal ring or situated in the posterior region of the buccal capsule, as the diagnostic characteristic for the definition of the genus *Denticamallanus*. The species reallocated here to *Denticamallanus*, except for *D. spiculastriatus* (=*Pr. spiculastriatus*), which was described in 2018 (Pinheiro et al., 2018), were initially proposed before the study of Moravec and Thatcher (1997). This timeline explains why these species, which all have tooth-like structures, are only now being reallocated.

*Denticamallanus rarus*, initially described in the genus *Spirocamallanus*, is the only species of *Denticamallanus* for which molecular data exist. Ailán-Choke et al. (2020) and Ailán-Choke and Pereira (2021) demonstrated by phylogenetic analysis of the 18S rDNA gene that the species forms a fully supported clade with *Sp. huacraensis* Ramallo, 2008.

*Spirocamallanus huacraensis*, described by Ramallo (2008), has spiral ridges in the buccal capsule of both sexes and 2 conical teeth only in females. Later, in a redescription, Ailán-Choke et al. (2019) noted that the species has ‘2–3 bladelike sclerotized structures arising between mid-length and bottom of the buccal capsule, present in both sexes’. These structures, described as either conical teeth or bladelike structures, are similar to the tooth-like structures found in *Denticamallanus*. This similarity suggests that the original authors misclassified *Sp. huacraensis,* and this grouping supports the maintenance of *Denticamallanus* as a valid genus.

**Figure 7.**
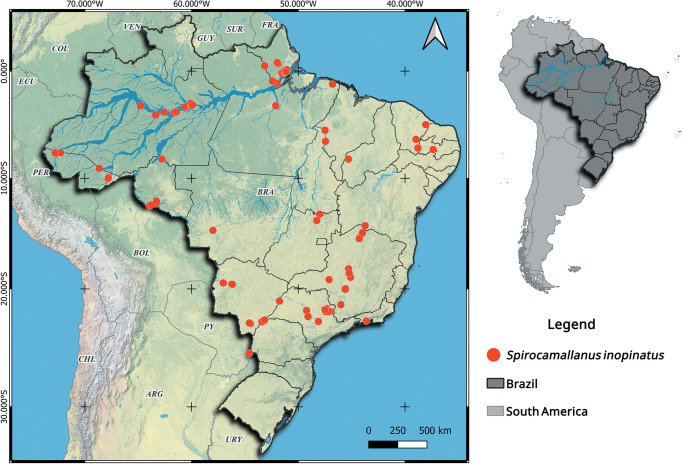
Geographical distribution of *Spirocamallanus inopinatus* in Brazil. Figure 8 long description.

*Spirocamallanus* is the most diverse camallanid genus in Brazil, with 16 species reported, followed by *Denticamallanus*, with 8 species, and *Serpinema*, with 7 species. *Spirocamallanus inopinatus* showed the widest geographic distribution, occurring in 18 Brazilian states across all 5 regions of the country (Fig. 7). It is also the species with the highest number of recorded hosts, parasitizing 144 host taxa, followed by *Sp. hilarii* (27 hosts), *Pr. peraccuratus* (21 hosts) and *D. iheringi* (19 hosts) (Fig. 5). We also highlight that 3 species of *Procamallanus* have been reported in Brazil. However, we reallocated 2 of these *Procamallanus* specie*s* to *Denticamallanus*, making *Pr. peraccuratus* the only species of the genus recorded in the country to date.

*Spirocamallanus inopinatus* is a species with low host specificity; besides parasitizing only fish, it can infect a wide range of host species across different host families and Brazilian biomes. Due to the wide diversity of hosts (Fig. 5) and locations (Fig. 7), the species shows considerable morphometric variation among specimens, yet maintains consistent morphological characteristics, as noted by Ailán-Choke et al. (2020). However, we cannot exclude the possibility that *Sp. inopinatus* represents a complex of cryptic species.

The geographical distribution pattern of *Sp. inopinatus* in freshwater fishes from Brazil was previously studied by Neves et al. (2020), who demonstrated that the species is widely distributed across Brazilian hydrographic basins, likely reflecting the broad distribution of its intermediate hosts (copepods) within these aquatic systems. The distribution of *Sp. inopinatus* in Brazil, illustrated in Figure 7, reinforces this wide-ranging pattern, suggesting that this camallanid is highly resistant to environmental changes, with a remarkable capacity for adaptation and use of diverse resources.

We also reallocated 1 species of *Camallanus* to *Serpinema*. The 2 genera are morphologically very similar, which can complicate species identification. *Serpinema emydidium*, initially described in the genus *Camallanus* by Mascarenhas and Müller (2017), exhibits characteristics of *Serpinema*, and therefore, we proposed its new combination.

*Camallanus, Serpinema* and *Zeylanema* have very similar buccal capsule morphology, which leads several authors to question the status of these genera (Moravec and Scholz, 1991; Moravec and Van As, 2015; Khadap, 2020). Saito et al. (2025) presented the first phylogenetic study using sequences of *Zeylanema* sp. The authors recovered a clade comprising species of all 3 genera, leading them to assume that *Serpinema* and *Zeylanema* should be considered subgenera of *Camallanus*.

However, Ailán-Choke and Pereira (2021) had previously shown that the monophyly of *Camallanus* spp. is not fully supported. Additionally, *Camallanus* and *Serpinema* (including *Paracamallanus*) together form a monophyletic clade, suggesting a correspondence between morphological features and phylogenetic and systematics relationships. We also conclude that the buccal capsule morphology is a reliable trait for differentiating these genera and that the current systematics of Camallanidae does not support the use of subgenera. Thus, we accept *Zeylanema* as a valid genus.

Molecular studies have questioned the validity of Camallanidae genera that rely solely on classification based on buccal capsule morphology, demonstrating that these characteristics do not reflect the family’s true phylogenetic relationships. Despite this, existing evidence still supports the genera *Spirocamallanus* and *Serpinema* (Harnoster et al. 2019; Ailán-Choke and Pereira, 2021), indicating that some morphological traits remain valid and valuable for classifying Camallanidae until additional studies are available. Furthermore, the buccal capsule morphology along camallanids might be a morphological trait that changed independently during the group’s evolution.

Analyses combining molecular and morphological data from Ailán-Choke and Pereira (2021) demonstrated that biogeography, habitat and host order strongly influence camallanid phylogeny. The morphological characteristics of the male tail were consistent in the authors’ analyses. For example, the presence or absence of a caudal alae, the pedunculate papillae and spicule symmetry frequently defined lineages in the study’s phylogenetic trees. Based on these findings, we consider the number, morphology and morphometry of the spicules, the number and distribution pattern of caudal papillae, and the presence or absence of caudal alae in males to be reliable morphological traits for identifying Camallanidae species.

Most camallanids reported from Brazil have been recorded in the Amazon and Atlantic Forest biomes, with 22 and 21 taxa, respectively. Among the Brazilian regions, the North (38 taxa) and Southeast (37 taxa) presented the highest diversity of camallanids. However, this apparent ‘equality’ in camallanid diversity between the 2 biomes and the 2 regions highlights the lack of studies on parasitic biodiversity in the Amazon region (which comprises the North of the country). While the Southeast (the Atlantic Forest is the predominant biome) comprises 4 states, the North includes 7 states, spanning a larger area and harbouring higher biodiversity. This scenario underscores the precarious state of taxonomy today, in which the description of new species is often treated as secondary rather than central to biodiversity studies (Ebach et al., 2011; Wägele et al., 2011). Finally, we highlight the urgent need to recognize taxonomy as a key field of knowledge and to promote the training of specialized scientists in the Amazon region.

The new key to the genera within the family Camallanidae is presented below, based on the main morphological characteristics documented for these nematodes. Our study contributes to understanding the diversity of camallanids in Brazil and assists future studies in identifying these nematodes.

### General diagnosis of Camallanidae Railliet and Henry, 1915

Parasites of gastrointestinal tract of amphibians, fish and reptiles. Body elongated. Oral opening rounded or dorsoventrally elongated. Lips absent. Buccal cavity well developed. Buccal capsule well-sclerotized, usually orange-brown, rounded or divided into 2 lateral valves. Oesophagus divided into 2 regions: anterior muscular and posterior glandular. Males typically with ventrally curved posterior end. Caudal alae usually present. Caudal papillae usually pedunculate in species with caudal alae. One or 2 spicules. Gubernaculum absent. Females didelphic. Opposed uterine branches. Ovoviviparous. Vagina directed to posterior region.

### Key to subfamilies of Camallanidae Railliet and Henry, 1915

1. Cup-like, continuous and rounded capsule, usually with posterior sclerotized basal ring. Tridents absent.........…………………………Procamallaninae (Fig. 1A)

2. Buccal capsule divided into 2 shell-like lateral valves, with longitudinal ridges and posterior sclerotized basal ring. Tridents present or absent……………………………………Camallaninae (Fig. 1B)

### Key to genera of the subfamily Procamallaninae Yeh, 1960

1a. Smooth buccal capsule, without ornamentations (ridges, punctuations or teeth) in both sexes...........………………………………………*Procamallanus* (Fig. 2A)

1b. Buccal capsule with ridges, punctuations or tooth-like structures………………………………………………………………2

2a. Buccal capsule with tooth-like structures or punctations.………………………………………………………3

2b. Buccal capsule with only ridges...............……………………..4

3a. Buccal capsule with tooth-like structures in at least 1 sex. Spiral ridges may be present………………*Denticamallanus* (Fig. 2B)

3b. Buccal capsule with punctations in both sexes……..…………..…………*Punctocamallanus* (Fig. 2C)

4a. Buccal capsule with spiral ridges...............……………………..5

4b. Buccal capsule with longitudinal ridges in both sexes…*Malayocamallanus* (Fig. 2D)

5a. Buccal capsule with only spiral ridges in both sexes……. *Spirocamallanus* (Fig. 2E)

5b. Smooth buccal capsule in males and buccal capsule with only spiral ridges in females…………………………………………*Spirocamallanoides* (Fig. 2F)

### Key to genera of the subfamily Camallaninae Yeh, 1960

1a. Buccal capsule composed of 3 chambers (first one is formed by 2 shell-like lateral valves), basal ring absent ………..…………………………………………2

1b. Buccal capsule composed of 2 shell-like lateral valves and a basal ring………………………………………….3

2a. Two first chambers with similar size………………………*Paracamallanus* (Fig. 3A)

2b. First chamber twice as large as the next 2.…………………..*Oncophora* (Fig. 3B)

3a. Tridents absent………………………*Neocamallanus* (Fig. 3C)

3b. Tridents present, associated laterally with valves……………………….……………4

4a. Trident with 1 branch…………………*Camallanides* (Fig. 3D)

4b. Tridents with 3 branches…………..……………………….5

5a. Buccal capsule lateral valves with saw-like longitudinal ridges…*Zeylanema* (Fig. 3E)

5b. Buccal capsule lateral valves with simple longitudinal ridges…………………………6

6a. Simple longitudinal ridges in 1 group………………………..*Camallanus* (Fig. 3F)

6b. Simple longitudinal ridges separated by a gap………………..…*Serpinema* (Fig. 3G)

## Supporting information

10.1017/S0031182026101668.sm001 Silva et al. supplementary material Silva et al. supplementary material

## Supplementary material

The supplementary material for this article can be found at https://doi.org/10.1017/S0031182026101668.

## Table 1 Long description

This table records the association between vertebrate host species and Camallanidae parasites in Brazil. It includes a wide range of fish families, such as Characidae, Cichlidae, and Pimelodidae, and their respective parasite species. Notably, Astyanax bimaculatus hosts eight different Camallanidae species, indicating a high diversity of parasitic relationships. Other significant hosts include Hoplias malabaricus and Pygocentrus nattereri, each with multiple parasite associations. The data suggests a trend of certain fish species being more susceptible to a variety of parasites, which may be due to ecological or biological factors. The table provides insights into the biodiversity and complexity of host-parasite interactions in Brazilian aquatic ecosystems.

Navigate back to Table 1.

## Figure 1 Long description

The image shows two sets of illustrations labeled A and B, depicting the buccal capsule morphology of subfamilies within the Camallanidae family. Set A represents Procamallaninae Yeh, 1960, with three views: apical, ventral and lateral. The apical view shows a round structure, while the ventral and lateral views depict elongated forms. Set B represents Camallaninae Yeh, 1960, also with apical, ventral and lateral views. The apical view shows a divided structure and the ventral and lateral views display elongated forms with internal ornamentations. Each view highlights distinct morphological features of the buccal capsule for each subfamily.

Navigate back to Figure 1.

## Figure 2 Long description

A shows Procamallanus with a simple capsule. B depicts Denticamallanus with internal arrow-like ornamentations. C illustrates Punctocamallanus with dotted patterns. D presents Malayocamallanus with vertical lines. E shows Spirocamallanus with spiral patterns. F depicts Spirocamallanoides with a smooth capsule. The bottom row shows male and female Spirocamallanoides with distinct capsule shapes.

Navigate back to Figure 2.

## Figure 3 Long description

Illustrative drawings depict the buccal capsule morphology of seven genera in the Camallaninae subfamily, shown in lateral view. Each drawing is labeled from A to G. A shows Paracamallanus, characterized by a distinct capsule shape. B illustrates Oncophora, with a unique capsule structure. C represents Neocamallanus, featuring specific morphological traits. D displays Camallanides, with its own capsule design. E shows Zeylanema, marked by particular features. F illustrates Camallanus, with a distinctive capsule form. G represents Serpinema, showcasing its unique morphology. Each genus has specific structural characteristics visible in the drawings.

Navigate back to Figure 3.

## Figure 4 Long description

Illustrations showing the morphology of tridents for different genera. A shows the trident for Camallanus, Serpinema and Zeylanema. B depicts the trident for Camallanides. C illustrates the trident for Oncophora. D shows the trident for Paracamallanus. Each illustration highlights the distinct shape and structure of the tridents associated with these genera.

Navigate back to Figure 4.

## Figure 5 Long description

A bar graph displays the number of hosts for different Camallanid taxa. The x-axis lists taxa names, while the y-axis shows the number of hosts, ranging from 0 to 144. Notable values include Spirocamallanus inopinatus with 144 hosts and Spirocamallanus pintoi with 50 hosts. A pie chart illustrates the diversity of Camallanid species, with segments labeled as 16 species, 8 species, 7 species, 3 species and single species for various genera. The chart uses different colors to represent each genus, including Denticamallanus, Procamallanus, Spirocamallanus, Camallanus, Oncophora, Paracamallanus and Serpinema.

Navigate back to Figure 5.

## Figure 6 Long description

A bar graph illustrating the number of host taxa of camallanids by host families in Brazil. The x-axis is labeled 'Host taxa by family' and the y-axis is labeled 'Number of host taxa'. The graph includes three categories: fishes, chelonians and serpentes. Fishes are represented by blue bars, chelonians by green bars and serpentes by red bars. The family Cichlidae has the highest number of host taxa among fishes, with 27 species. Anostomidae follows with 23 taxa and Pimelodidae with 21 taxa. Among chelonians, the family Chelidae has 6 host species. Viperidae is the only snake family represented, with 1 species recorded. Each family is labeled along the x-axis, with varying heights of bars indicating the number of host taxa for each family.

Navigate back to Figure 6.

## Figure 7 Long description

A map illustrating the geographical distribution of Spirocamallanus inopinatus in Brazil, marked by red dots across various locations. The map includes major rivers and state boundaries. An inset map of South America highlights Brazil. A scale bar and compass rose are present for reference.

Navigate back to Figure 7.
